# Two Horizontally Transferred Xenobiotic Resistance Gene Clusters Associated with Detoxification of Benzoxazolinones by *Fusarium* Species

**DOI:** 10.1371/journal.pone.0147486

**Published:** 2016-01-25

**Authors:** Anthony E. Glenn, C. Britton Davis, Minglu Gao, Scott E. Gold, Trevor R. Mitchell, Robert H. Proctor, Jane E. Stewart, Maurice E. Snook

**Affiliations:** 1 USDA, ARS, Richard B. Russell Research Center, Toxicology & Mycotoxin Research Unit, Athens, Georgia, United States of America; 2 University of Georgia, Department of Plant Pathology, Athens, Georgia, United States of America; 3 USDA, ARS, National Center for Agricultural Utilization Research, Mycotoxin Prevention and Applied Microbiology Research Unit, Peoria, Illinois, United States of America; 4 Colorado State University, Bioagricultural Sciences & Pest Management, Fort Collins, Colorado, United States of America; The University of Wisconsin—Madison, UNITED STATES

## Abstract

Microbes encounter a broad spectrum of antimicrobial compounds in their environments and often possess metabolic strategies to detoxify such xenobiotics. We have previously shown that *Fusarium verticillioides*, a fungal pathogen of maize known for its production of fumonisin mycotoxins, possesses two unlinked loci, *FDB1* and *FDB2*, necessary for detoxification of antimicrobial compounds produced by maize, including the γ-lactam 2-benzoxazolinone (BOA). In support of these earlier studies, microarray analysis of *F*. *verticillioides* exposed to BOA identified the induction of multiple genes at *FDB1* and *FDB2*, indicating the loci consist of gene clusters. One of the *FDB1* cluster genes encoded a protein having domain homology to the metallo-β-lactamase (MBL) superfamily. Deletion of this gene (*MBL1*) rendered *F*. *verticillioides* incapable of metabolizing BOA and thus unable to grow on BOA-amended media. Deletion of other *FDB1* cluster genes, in particular *AMD1* and *DLH1*, did not affect BOA degradation. Phylogenetic analyses and topology testing of the *FDB1* and *FDB2* cluster genes suggested two horizontal transfer events among fungi, one being transfer of *FDB1* from *Fusarium* to *Colletotrichum*, and the second being transfer of the *FDB2* cluster from *Fusarium* to *Aspergillus*. Together, the results suggest that plant-derived xenobiotics have exerted evolutionary pressure on these fungi, leading to horizontal transfer of genes that enhance fitness or virulence.

## Introduction

Land plants can provide carbon-rich habitats for microbes in ecologically diverse geographic ranges such that many fungi and bacteria inhabit the interior of healthy plants as endophytes for most if not all of their life cycle, and typically do so without causing disease. However, under some environmental or physiological conditions, microbes that are typically endophytic can become pathogenic [1, 2]. Schulz and Boyle [1] refer to this “endophytic continuum” as a balance of antagonisms that can shift depending on virulence traits and plant defense responses. Maintaining the balance results in asymptomatic colonization.

Defense responses of plants often include *de novo* production of metabolites (phytoalexins) or the release of preformed compounds (phytoanticipins) that can inhibit growth of pathogenic microbes [3]. Maize, wheat, and rye produce the two benzoxazinone phytoanticipins 2,4-dihydroxy-7-methoxy-1,4-benzoxazin-3-one (DIMBOA) and 2,4-dihydroxy-1,4-benzoxazin-3-one (DIBOA) [4]. Due to their inherent instability once released, DIMBOA and DIBOA spontaneously degrade to the more stable corresponding benzoxazolinones, 6-methoxy-benzoxazolin-2-one (MBOA) and 2-benzoxazolinone (BOA), respectively. Collectively we refer to these four metabolites as Bx compounds, which deter insect herbivory and are implicated in resistance to various fungal and bacterial diseases [4].

**Fig 1:**
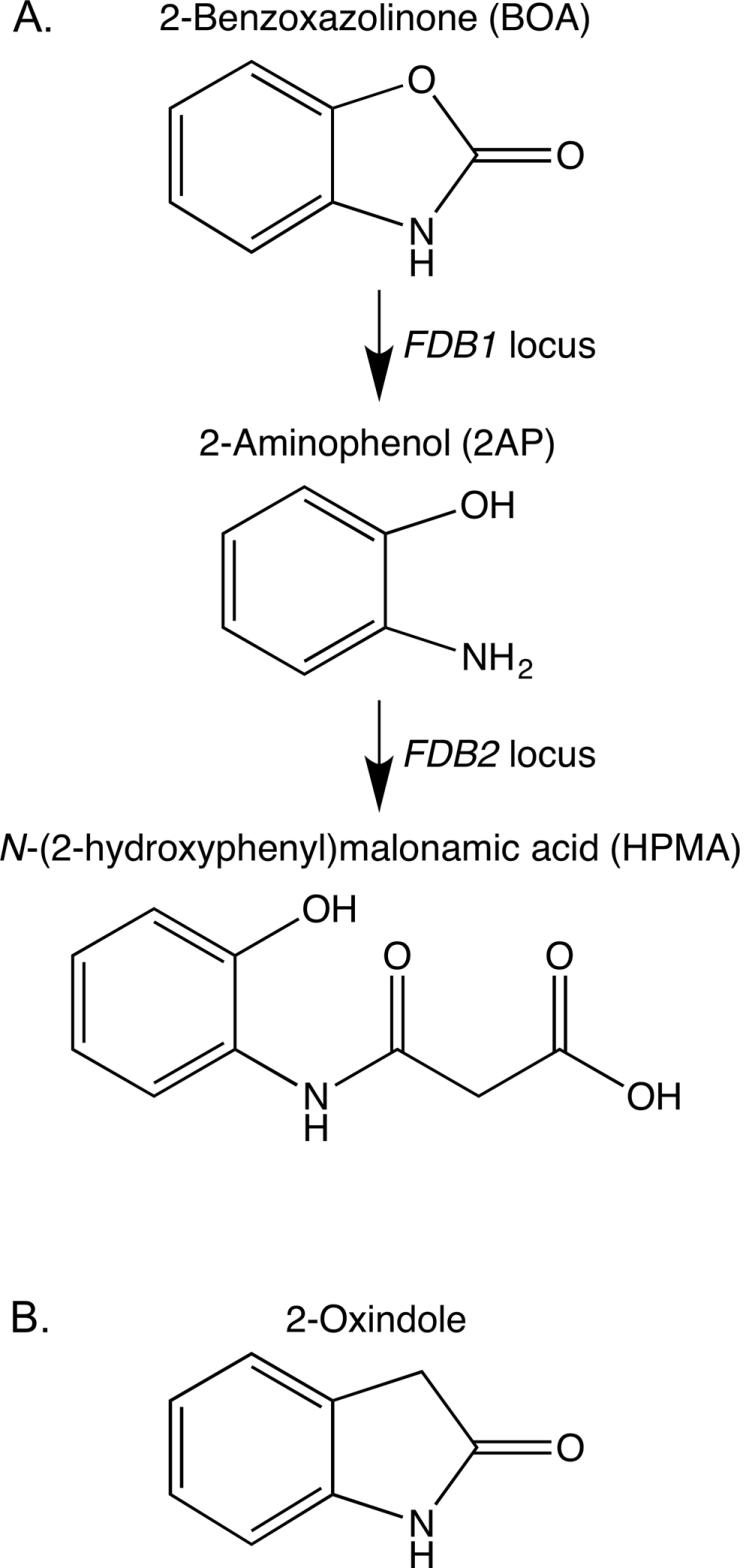
Detoxification pathway of 2-benzoxazolinone (BOA), and structure of a compound similar to BOA, 2-oxindole. (A) The *Fusarium verticillioides* pathway for metabolic detoxification of BOA into *N*-(2-hydroxyphenyl)malonamic acid. The *FDB1* locus facilitates hydrolysis of BOA to produce 2-aminophenol (2AP), while the *FDB2* locus facilitates modification of 2AP by addition of a malonyl group to form HPMA. (B) 2-Oxindole, a γ-lactam compound structurally similar to BOA.

Several *Fusarium* species can tolerate MBOA and BOA at concentrations that inhibit the growth of other fungi [4–6]. Tolerance results from hydrolysis of the five-membered oxazole ring of MBOA and BOA and loss of the carbonyl group (Fig 1), followed by an additional modification that yields the non-toxic metabolites *N*-(2-hydroxy-4-methoxyphenyl) malonamic acid (HMPMA) and *N*-(2-hydroxyphenyl) malonamic acid (HPMA), respectively [7–9]. MBOA and BOA are structurally interesting since the oxazole ring has moieties for both a γ-lactone and a γ-lactam (Fig 1). Only a limited number of *Fusarium* species are known to detoxify MBOA or BOA, with the maize pathogens *Fusarium verticillioides* and *Fusarium subglutinans* being among the most tolerant [5, 6, 10, 11]. Most Bx-tolerant *Fusarium* species are associated with maize and wheat.

*Fusarium verticillioides* requires two meiotically defined loci, *FDB1* and *FDB2*, for tolerance and detoxification of the benzoxazolinones (Fig 1) [8]. The loci are not linked, and functional alleles are required at both loci for metabolism of BOA to HPMA. We previously determined the *FDB2* locus is a gene cluster of at least 13 genes located on chromosome 3 [7]. Further, the *FDB2* cluster gene, *NAT1*, encodes a member of the arylamine *N*-acetyltransferase (NAT) family and is essential for transformation of 2-aminophenol (2AP) to HPMA [7]. Nat1 is unique for the NAT family in that it is a *N*-malonyltransferase with specificity for malonyl-CoA instead of acetyl-CoA [12]. We originally designated this gene *FDB2* but have recently adopted the designation *NAT1* to more clearly associate it with the NAT family and to facilitate clearer annotation of other NAT paralogs in *F*. *verticillioides*. NATs are found in organisms ranging from bacteria to humans but are not found in plants [13, 14]. We have noted a number of homologs in fungi, but *NAT1* orthologs appeared to be restricted to a narrow range of genera containing pathogens of maize and wheat [12, 13].

Recently, deletion analysis in *Fusarium pseudograminearum* indicated its *NAT1* ortholog, which the authors designated *FDB2*, also is required for tolerance and metabolism of MBOA and BOA [10]. Interestingly, *NAT1* was required for full virulence since Bx-sensitive *Δnat1* mutants of *F*. *pseudograminearum* exhibited significant reduction in wheat head blight severity [10]. In contrast, while deletion analysis indicated *NAT1* in *F*. *verticillioides* is required for Bx-tolerance [7], such tolerance is not required for virulence of *F*. *verticillioides* on maize seedlings [8]. Yet, the metabolic detoxification of Bx compounds was shown to be a major factor contributing to the dominance of Bx tolerant fungi in maize field environments, thus providing an ecological advantage by increasing fitness for niche competition [6, 15]. The enhanced fitness and persistence of *F*. *verticillioides* is of major economic and food safety concern due to reduced grain quality from maize ear rot and elevated levels of the fumonisin mycotoxins in the kernels. Fumonisin B1 (FB1) causes liver and renal carcinogenicity in laboratory rodents [16] and is noted as a possible cause of cancer, childhood stunting, and neural tube birth defects in humans [17, 18]. FB1 also causes animal diseases, most notably leukoencephalomalacia in horses and pulmonary edema in pigs [16, 19]. FB1 is also a phytotoxin and contributes to the virulence of *F*. *verticillioides* toward susceptible maize genotypes [20–22], thus being a factor in the “balance of antagonisms” observed for this host-fungus association.

Our previous determination of the *FDB2* locus as a gene cluster was based in part on transcriptional profiling, yet *NAT1* was the only gene within the cluster that was essential for Bx-tolerance [7]. A 9-gene *NAT1* containing cluster was also delineated in *F*. *pseudograminearum* based on reverse transcriptase-quantitative PCR [10]. As for the *FDB1* locus in *F*. *verticillioides*, its molecular genetic nature has not been previously evaluated to determine if it consists of a single gene or a cluster of genes like the *FDB2* locus. A gene designated *FDB1* was recently characterized in *F*. *pseudograminearum* and *Fusarium graminearum* and demonstrated to be essential for hydrolysis of BOA [23], the first step in the detoxification pathway resulting in production of the intermediate 2AP (Fig 1). The encoded protein (Fdb1) had domain homology to the class B metallo-β-lactamase superfamily, but instead of being a typical β-lactamase that hydrolyzes β-lactams such as penicillin and other similar antibiotics [24], this *Fusarium* protein was categorized as a γ-lactamase because of the γ-lactam structure of BOA. Kettle et al. [23] were functionally justified in designating this gene *FDB1* due to the encoded hydrolysis of BOA to 2AP, but the gene is adjacent to *NAT1* in the 9-gene *F*. *pseudograminearum* cluster that is orthologous to the *FDB2* cluster of *F*. *verticillioides*. Thus, linkage of the *F*. *pseudograminearum FDB1* and *FDB2/NAT1* genes is in contrast with the unlinked, meiotically defined *FDB1* and *FDB2* loci of *F*. *verticillioides*.

Here we extend the characterization of *FDB* loci with a detailed analysis of the *F*. *verticillioides FDB1* locus, which like the *FDB2* locus consists of a gene cluster. The *FDB1* cluster was defined by coexpression of nine contiguous genes when *F*. *verticillioides* was exposed to BOA. As with *F*. *pseudograminearum* and *F*. *graminearum*, a metallo-β-lactamase within the *FDB1* cluster was essential for detoxification of BOA. Instead of denoting this gene as *FDB1*, we designate it as *MBL1* in order to more clearly convey the encoded enzymatic activity and to aid in systematic annotation of additional metallo-β-lactamase homologs encoded in the *F*. *verticillioides* genome. While β-lactamases are well studied in bacteria for their impact on antibiotic resistance, these are the first detailed studies of hydrolytic lactamases in fungi. We further demonstrate that only three of the 49 *Fusarium* species examined had both an *FDB1* and *FDB2* cluster, whereas 34 species had a complete or partial *FDB2* cluster only, and 12 species lacked both clusters. None of the *Fusarium* species had a *FDB1* cluster only. Lastly, protein phylogenies suggested independent horizontal gene transfer (HGT) of the two *FDB* gene clusters. One involved transfer of the *FDB1* cluster from *Fusarium* to *Colletotrichum*, and another was transfer of the *FDB2* cluster from *Fusarium* to *Aspergillus*. These results suggest plant-derived xenobiotics may have exerted evolutionary pressure on these fungi, leading to horizontal transfer of genes that enhance fungal fitness or virulence when associated with Bx-producing plants.

## Materials and Methods

### Fungal strains and growth conditions

Strains of *F*. *verticillioides* were grown and maintained as previously described [5, 8]. Wild-type strain FRC M-3125 (*FDB1*/*FDB2*) and mutant AEG 74-A4-3 (*fdb1*/*FDB2*) were used for genetic characterization of the *FDB1* locus. Glenn et al. [8] provides additional details on strain AEG 74-A4-3. The fungi were grown routinely using potato dextrose agar (PDA; Sigma-Aldrich Chemical Co., Milwaukee, WI, USA) and potato dextrose broth (PDB; Sigma-Aldrich Chemical Co.). Stock solutions of BOA (100 mg ml^-1^; Sigma-Aldrich Chemical Co.) were prepared in ethanol, and strains were assessed for tolerance to BOA at 0.9 mg ml^-1^ final concentration on BOA medium [5]. Structurally similar 2-oxindole (Sigma-Aldrich Chemical Co.) was also assessed in the same manner as BOA. All such cultures, including the controls, contained 1% ethanol.

Growth curve analysis was conducted using a Bioscreen C automated system (Growth Curves USA, Piscataway, NJ, USA). Strains were inoculated in PDB (2000 conidia in 200 μL PDB per well) using the Bioscreen honeycomb microtiter plates. The wells were amended with BOA (0.5 mg ml^-1^). Each treatment was replicated in five wells. The microtiter plates were incubated four days at 28C with constant shaking and OD_600_ measurements taken every 30 min. To simplify presentation of the growth curves, only measurements taken every two hours were graphed. The experiment was conducted twice.

### Assessment of BOA degradation and HPMA production

Strains were screened for metabolism of BOA into HPMA using the agar plug thin-layer chromatography (TLC) method of Glenn et al. [5]. In brief, strains were grown for 7–14 days in 24-well culture plates, each well containing 1.5 ml of BOA agar medium, and metabolism of BOA was assessed by taking a single plug from one well using a no. 5 cork borer and spotting the plug on a TLC sheet (Whatman Ltd., Maidstone, Kent, England; silica gel coating, 254-nm UV indicator, aluminum backing). The TLC sheets were developed in a saturated chamber containing toluene-ethyl acetate-formic acid (50:40:10,vol/vol/vol). Developed sheets were dried and photographed under UV light (254 nm) using an Alpha Innotech FluorChem 8000 digital imaging system (San Leandro, CA, USA). HPMA standard was available from previous analyses [5, 9].

For HPLC evaluation of BOA detoxification and production of metabolites, the strains were grown for three days in 50 mL PDB on a rotary shaker (200 rpm) at 27C. Duplicate cultures were treated with 50 μg/mL of either ethanolic BOA or 2-oxindole. Control duplicates were treated with 1% ethanol only. Uninoculated PDB was also amended with BOA, 2-oxindole, and ethanol to serve as chromatographic controls. Cultures were incubated an additional six hours before centrifugation to collect the supernatants, which were kept frozen until ready for analysis. Thawed supernatants were filtered (0.45 μm nylon) and examined (20 μL) without dilution on Agilent Technologies 1200 series HPLC system (Walbronn, Germany) with a Beckman Ultrasphere 5 μm ODS column (250 mm x 4.6 mm) and a diode array detector monitoring 282 nm (20 nm bandwidth) using 550 nm (100 nm bandwidth) as a reference spectrum. Compounds were separated with a flow rate of 1.0 mL/min and gradient elution using mobile phases consisting of 10% methanol with 0.1% phosphoric acid (A) and 100% methanol with 0.1% phosphoric acid (B). The gradient was 100% A to 100% B from 0 to 35 min, then held at 100% B for 12 min before cycling back to 100% A for the next sample. Compounds in culture supernatants were identified by comparison of retention times to the standards of BOA and HPMA (each at 0.1 μg/μL).

### Microarray analysis

Wild-type *F*. *verticillioides* FRC M-3125 was grown for three days in PDB (50 ml) at 200 rpm in the dark at 27C. The fungus was transferred (1 ml) to replicate flasks of fresh PDB (50 ml) and grown for three more days to synchronize cultures. For induction, ethanol-dissolved BOA (50 μg ml^-1^ final conc.; 1% ethanol) was added to cultures, while only ethanol (1%) was added for the uninduced treatment. After 2 hr incubation, the fungal growth was harvested by vacuum filtration, flash frozen in liquid nitrogen, and stored at -80C until ready for extraction. Samples were ground with liquid nitrogen, and RNA was extracted with Ambion PureLink RNA Mini Kit (Life Technologies, Carlsbad, CA, USA) using homogenizers, followed by treatment with Turbo DNase (Life Technologies), all according to manufacturer’s instructions. Double stranded cDNA was generated using Roche cDNA Synthesis System (Indianapolis, IN, USA), again according to instructions, along with additional recommendations from Roche NimbleGen (Madison, WI, USA). Roche NimbleGen (Iceland) conducted microarray hybridizations, data acquisition, and initial analysis. Microarrays were designed based on genomic and EST datasets as previously described [25, 26]. Normalized data were compared using DNASTAR ArrayStar v5.0 analysis software (Madison, WI, USA). The experiment was conducted with two biological replications.

### Gene deletion and complementation

Deletion constructs for FVEG_08289 (*AMD1*), FVEG_08290 (*DLH1*), and FVEG_08291 (*MBL1*) were made using the double-joint PCR procedure [27]. Table 1 summarizes the strains created for this study. Each gene-coding region was targeted for deletion in strain FRC M-3125 by transformation and homologous recombination with a construct containing the hygromycin resistance cassette flanked by *F*. *verticillioides* genomic sequence. To maintain consistency, references to 5’ and 3’ when amplifying gene flanks is not based on the transcriptional orientation of each gene but is instead based on the plus strand genomic sequence of the *FDB1* gene cluster as provided by the Broad Institute *Fusarium* Comparative Database (http://www.broadinstitute.org/annotation/genome/fusarium_group/MultiHome.html). During the first round of PCR, the 5’ and 3’ flanking sequences were amplified, respectively, with primer pairs AMD1-5’outer/AMD1-5R (1254 bp) and AMD1-3F/AMD1-3’outer (1618 bp), DLH1-5’outer/DLH1-5R (1691 bp) and DLH1-3F/DLH1-3’outer (1164 bp), and MBL1-5’outer/MBL1-5R (1370 bp) and MBL1-3F/MBL1-3’outer (1172 bp) (S2 Table; S9 Fig). The hygromycin gene cassette (1585 bp) was amplified from plasmid pCB1003 with primer pair M13Fv2/M13Rv2 (S2 Table). Reactions consisted of 1.0 unit of Platinum *Taq* DNA polymerase (Invitrogen, Carlsbad, CA, USA), 1× PCR buffer, 2.5 mM MgCl_2_, 200 μM each dNTP, 0.2 μM forward primer, 0.2 μM reverse primer, and 10 ng of template DNA. Thermal cycling consisted of initial denaturation at 95C for 2 min; followed by 40 cycles of 95C for 30 s, 56C for 30 s, and 72C for 2 min; and a final incubation at 72C for 8 min.

**Table 1:** Strains used and created in this study.

Strain	RRC Strain #	Genotype	Description
FRC M-3125^a^	N/A	Wild type	Strain used for targeted deletions
AEG 74-A4-3^b^	RRC 1130^c^	*fdb1/FDB2*	Possesses *fdb1* mutant allele
M3125::Δ08289–2	RRC 2022	*Δamd1*	Deletion of *AMD1*, FVEG_08289
M3125::Δ08290–11	RRC 2033	*Δdlh1*	Deletion of *DLH1*, FVEG_08290
M3125::Δ08291–33	RRC 2041	*Δmbl1*	Deletion of *MBL1*, FVEG_08291
M3125::08291–34	RRC 2042	*MBL1* ectopic	Ectopic insertion
M3125::08291–35	RRC 2043	*MBL1* ectopic	Ectopic insertion
M3125::Δ08291–36	RRC 2044	*Δmbl1*	Deletion of *MBL1*, FVEG_08291
Δ08291–36::C2	RRC 2057	*Δmbl1*::*MBL1*	*Δmbl1* complemented with *MBL1*

^a^FRC, *Fusarium* Research Center, Pennsylvania State University.

^b^AEG, Anthony E. Glenn [8].

^c^RRC, Richard B. Russell Research Center.

Second round fusion PCR was performed for each gene by combining both flanking regions and the hygromycin cassette in a 1:1:4 ratio with PCR components as noted above but without addition of primer. Cycling parameters were 95C for 2 min; followed by 15 cycles of 95C for 10 s, 58C for 2 min, and 72C for 5 min; and final incubation at 72C for 10 min. For the third round PCR, multiple 50 μL reactions were performed using 0.5 μL of each second round fusion reaction as template for amplification of the final deletion constructs using the respective nested primer pairs: AMD1-5F/AMD1-3R, DLH1-5F/DLH1-3R, and MBL1-5F/MBL1-3R (S2 Table; S9 Fig). Third round cycling parameters were initial denaturation at 95C for 2 min; followed by 35 cycles of 95C for 10 s, 58C for 10 s, and 72C for 4 min; and final incubation at 72C for 8 min. The ≥3.5 kb amplicons were gel-purified and used directly for transformations [21]. Single-spore purified hygromycin-resistant transformants were screened by PCR for gene deletions using the respective 5’outer primer and a primer within the hygromycin cassette (primer HygRev for *AMD1* and *DLH1* transformants; HygFor for *MBL1* transformants) (S2 Table). Likewise, the respective 3’outer primer was paired with either primer HygFor (*AMD1* and *DLH1*) or HygRev (*MBL1*). Deletion and ectopic strains were confirmed by Southern hybridization using a flanking region of each respective gene as probe. Probes were amplified as noted in the following section. Gene deletion strains were assessed for their ability to grow on BOA-amended medium as noted above.

The wild-type *MBL1* gene was amplified with primers MBL1-5F and MBL1-3’outer to encompass the 1200-bp ORF plus 1230 bp upstream of the predicted start codon and 1078 bp downstream of the stop codon for a total amplicon of 3508 bp (S9 Fig). Amplification parameters were 95C for 5 min; followed by 40 cycles of 95C for 10 s, 56C for 10 s, and 72C for 3.5 min; and final incubation at 72C for 10 min. The amplicon was cleaned (QIAquick PCR purification kit, Qiagen, Inc., Valencia, CA, USA) and combined with undigested pGEN-NotI (1 μg of each) for PEG-mediated cotransformation of protoplasts generated from the Δ*mbl1* deletion strain. Transformants were selected on hygromycin B (150 μg ml^-1^; Roche Diagnostics, Indianapolis, IN, USA) and geneticin (200 μg ml^-1^; Sigma-Aldrich Chemical Co.). To assess for complementation they were screened by gene-specific PCR with primers MBL1for and MBL1rev (S2 Table) and for tolerance to BOA (0.9 mg ml^-1^). Cycling parameters for the 528 bp amplicon were 95C for 5 min; 40 cycles of 95C for 10 s, 60C for 10 s, and 72C for 30 s; and final extension of 72C for 2 min. BOA-tolerant transformants were also evaluated for the wild-type *MBL1* gene by Southern hybridization.

### Southern hybridization

Genomic DNA of *F*. *verticillioides* was extracted from 4- to 7-day-old PDB liquid cultures using the DNeasy Plant Mini Kit (Qiagen Inc.) following the manufacturer’s protocol. Plasmids and cosmids were extracted from *Escherichia coli* cultures using the QIAprep Spin Miniprep Kit and Large-Construct Kit (Qiagen Inc.), respectively. Southern hybridizations were performed by blotting 5 μg digested DNA from a 1.0% agarose Tris-acetate-EDTA gel onto a Hybond-N+ nylon membrane (Amersham Biosciences, Buckinghamshire, England) according to standard procedures. The respective restriction digests were SalI for *AMD1*, HindIII for *DLH1*, and EcoRI for *MBL1*. Gene-specific probes were generated using a digoxigenin (DIG) PCR labeling kit (Roche, Indianapolis, IN, USA). The respective primer pairs were AMD1-5F/FvAm4 for *AMD1*, DLH1-5F/FvAm18 for *DLH1*, and MBL1-5F/DLH1-3R for *MBL1* (S2 Table). Each probe (120 μl) was diluted in 15 ml of DIG Easy Hyb solution (Roche) and heated to 100C for 5 min. Hybridization and chemiluminescent detection of membranes were performed using Roche solutions and recommended procedures. The Alpha Innotech FluorChem 8000 digital imaging system was used for visualization. Exposures of 50 min typically were required.

### Cosmid identification and complementation of an *fdb1* mutation

In addition to the microarrays, we previously described the creation of a suppression subtractive hybridization (SSH) library of *F*. *verticillioides* genes differentially expressed when the fungus was exposed to BOA [7]. A putative amidase (*AMD1*, FVEG_08289) was well represented in the SSH library and was targeted to facilitate isolation of a cosmid from a *F*. *verticillioides* cosmid library [7]. Two probes targeting different regions of the amidase cDNA were individually hybridized to cosmid macroarrays to positively identify candidate clones. The probes were generated using primer pairs 1B8For/DLH1-5F and 4B3For/4B3Rev based on the thermal cycling procedure noted above. Both probes hybridized to cosmid F5D9, which was subjected to transposon insertion mutagenesis using the EZ-Tn5 <KAN-2> Insertion Kit (EPICENTRE Biotechnologies, Madison, WI, USA) based on the manufacturer’s instructions. The insertion clones were sequenced and contigs assembled using Sequencher v.5.0 (Gene Codes Corp., Ann Arbor, MI, USA). The clones having single transposon insertions within the *F*. *verticillioides* genomic DNA carried on the cosmid were used in a functional complementation assay. Insertion clones and the original F5D9 cosmid were individually transformed into protoplasts of *F*. *verticillioides* strain AEG 74-A4-3 as previously described [7, 21]. Hygromycin-resistance was conferred by the cosmid, and transformants were single-spore isolated and assessed for their ability to grow on 0.9 mg ml^-1^ BOA agar medium after 7–14 days incubation at 27C in the dark.

### Quantitative real-time PCR (qPCR)

Conidia (approx. 3 × 10^7^) from a 3-day-old PDB culture of strain FRC M-3125 were inoculated into fresh PDB (50 ml), grown for an additional three days, and then amended with either ethanolic BOA (50 μg ml^-1^ final conc.; 1% ethanol) or 1% ethanol alone. Cultures were harvested by vacuum filtration after 0.5 h incubation at 27C in the dark. After grinding fungal samples in liquid nitrogen, total RNA was extracted and treated with DNase I as described above for microarrays. Treated RNA was cleaned using RNeasy MinElute Cleanup Kit (Qiagen Inc.) and assessed for RNA integrity on an Agilent 2100 Bioanalyzer (Waldbronn, Germany). The Clontech QTaq One-Step qRT-PCR SYBR Kit (Mountain View, CA, USA) was used for qPCR assays. Each 25 μl reaction consisted of 1X Clontech One-Step qRT-PCR Buffer plus SYBR, 1X QTaq DNA Polymerase Mix, 1X qRT Mix, 0.15 μM forward primer, 0.15 μM reverse primer, and 30 ng of RNA. Control reactions were performed by adding RNase/DNase free water instead of RNA template. The reverse transcriptase mix was excluded from several runs to verify the RNA was not contaminated with genomic DNA. Primers for *TUB2* (TUB2-F and TUB2-R) were described by Choi and Shim (2008). Forward and reverse qPCR primers for *AMD1*, *DLH1*, and *MBL1* are listed in S2 Table. The cycling parameters were: 1 cycle of 48C for 20 min for first-strand cDNA synthesis, 1 cycle of 95C for 3 min for activation of QTaq DNA polymerase, and then 45 cycles of 95C for 15 s and 65C for 1 min (optics on). Melt curve analysis was conducted to eliminate erroneous results (60-90C; 0.2C per s). Reactions were performed using a Cepheid Smart Cycler (Sunnyvale, CA, USA). The cycle threshold (Ct) was determined for each sample based on second derivative curve analysis and a manual threshold of 30 fluorescence units. Expression of the three genes was normalized to *TUB2* expression (endogenous reference) and quantified relative to the untreated control based on the comparative Ct method as described in Applied Biosystems User Bulletin #2. The qPCR experiment was conducted twice with a minimum of three technical replications per biological replicate.

### Virulence assays

Seeds of sweet corn Silver Queen (W. Atlee Burpee & Co., Warminster, PA, U.S.A.) were surface sterilized, imbibed, and heat shocked as previously described [21]. For each fungal strain, 50 seeds were placed in a standard petri dish (100 x 15 mm) with 10 mL of spore suspension (1x10^4^ spores/mL in water). Uninoculated control seed received 10 mL of sterile water. After overnight incubation in the dark at 27C, seed were planted in autoclaved potting mix in 4-inch azalea pots. Three pots of 10 seed/pot were prepared for each treatment. Pots were placed in plastic saucers and watered from below by allowing water to soak into the soil through the bottom of the pots. Pots were watered from the bottom on days 2, 4, and 6 after planting. All subsequent watering was as needed and added to the soil from the top. Plants were grown for 14 days in a growth chamber having 16-hr days at 30C and 8-hr nights at 20C (light and temperature were programmed to increase/decrease gradually over 2-hr intervals with 30-min segments). Seedlings were visually inspected for leaf lesions, cut at the soil line, and aerial tissues were freeze-dried to determine dry weight.

### Fumonisin production assay

Each strain (1x10^7^ spores) was inoculated onto 5 g of autoclaved cracked maize kernels in 20-mL scintillation vials as previously described [21]. Triplicate vials were prepared for each strain and incubated for 7 days at 27C in the dark. The cultures were extracted by adding 10 mL of acetonitrile:water (1:1) with 5% formic acid to each vial, shaking vigorously to break up the colony, and then shaking gently for 3 h on a rotary shaker. Extracts were diluted 1000-fold in acetonitrile:water (3:7) with 1% formic acid, filtered (0.45 μm nylon), and analyzed by liquid chromatography-mass spectrometry for fumonisin B1, B2, and B3 [28].

### Genome sequences of *Fusarium* species

For all genome sequencing of *Fusarium* species done as part of the current study, genomic DNA was purified with the ZR Fungal DNA MiniPrep Kit (Zymo Research). For most species, genome sequence data were generated at the USDA National Center for Agricultural Utilization Research using either an Ion Torrent or Illumina MiSeq sequencing platform. Genomic DNA libraries for Ion Torrent sequencing were constructed from 1 μg of purified DNA using the NEBnext Fast DNA Library Kit (New England Biolabs) and data were generated with the Ion Torrent 400-bp template kit and the HiQ sequencing chemistry on a 318v2 chip. Genomic DNA libraries for Illumina MiSeq sequencing were constructed from 1 ng of purified DNA using the Nextera XT DNA Library Preparation Kit (Illumina). For the MiSeq platform, sequencing data were obtained using the MiSeq Reagent Kit v3. Reads generated from either platform were trimmed and assembled with CLC Genomics Workbench version 8.0 (CLC bio-Qiagen). Genome sequence data for some species were generated with the Illumina HiSeq 2000 sequencing platform and assembled by the Beijing Genome Institute-Hong Kong.

To identify *FDB1* and *FDB2* loci in the genome sequences, individual *F*. *verticillioides FDB* cluster gene sequences were used to query genomic sequence databases using the BLASTn application in CLC Genomics Workbench. Manual annotation of cluster genes was done by comparison to *F*. *verticillioides* and other annotated species and by using FGENESH [29] through www.softberry.com.

### Phylogenetic evaluation of the *FDB1* and *FDB2* clusters

Encoded proteins common to both the *FDB1* and *FDB2* clusters were used to identify homologous accessions within the National Center for Biotechnology Information (NCBI) non-redundant database using BLASTp. Each gene was also used for BLASTn searches of *Fusarium* genomes as noted above. Genomic sequences with high identity (typically E-value = 0.0) were of particular interest if orthologous *FDB1* and/or *FDB2* gene clusters were evident. Predicted protein sequences were aligned using MUSCLE and phylogenies were inferred from the alignment using MrBayes [30] and PhyML [31], all implemented through Geneious v.7.1.9 (Biomatters Ltd., Auckland, New Zealand). Alignments were evaluated using MEGA v.6.06 [32] to determine the maximum likelihood substitution model having the best fit. The Whelan and Goldman (WAG) model with estimated proportion of invariable sites and gamma distribution was selected for analyses of each dataset. MrBayes parameters were the following: 10,000,000 chain length; 4 heated chains; 0.2 heated chain temp; 200 subsampling frequency; 0.125 burn-in length. Bootstrap branch support of the PhyML analysis was assessed based on 200 replications.

To further validate the inferences of HGT of the *FDB1* and *FDB2* gene clusters, a phylogenetic topological congruence test was conducted for the amino acid derived phylogenies. The Shimodaira-Hasegawa test (SH test), was performed on maximum likelihood phylogenies conducted using the Phangorn package [33] using 1000 RELL resampling replicates implemented in R v3.2.2 (R Foundation for Statistical Computing, Vienna, Austria). Seven phylogenies from the *FDB1* or *FDB2* clusters were tested for topological congruence against a species tree that was estimated using the elongation factor 1 alpha (TEF) locus, which is commonly used to identify species within *Fusarium* [34]. To conduct the SH test, the compared topologies must include the same isolates, therefore pairwise tests were conducted individually for the seven *FDB* phylogenies against a TEF species tree containing the same sampling of species.

### Public availability of data

Microarray data were deposited in NCBI's Gene Expression Omnibus (GEO) and are accessible through GEO Series accession GSE74832 (http://www.ncbi.nlm.nih.gov/geo/). The annotated genomic sequences of *F*. *camptoceras* NRRL 13381 (*FDB1* and *FDB2* gene clusters), *Fusarium proliferatum* NRRL 62905 (*FDB2* gene cluster), and *F*. *subglutinans* NSM 107 (*FDB1* and *FDB2* gene clusters) are available as NCBI GenBank accessions KU055633 through KU055637, respectively. The additional unpublished sequence data for *FDB* loci and housekeeping genes used in this study are available from R.H. Proctor.

## Results

### *Fusarium verticillioides* genes differentially expressed in response to BOA exposure

**Fig 2:**
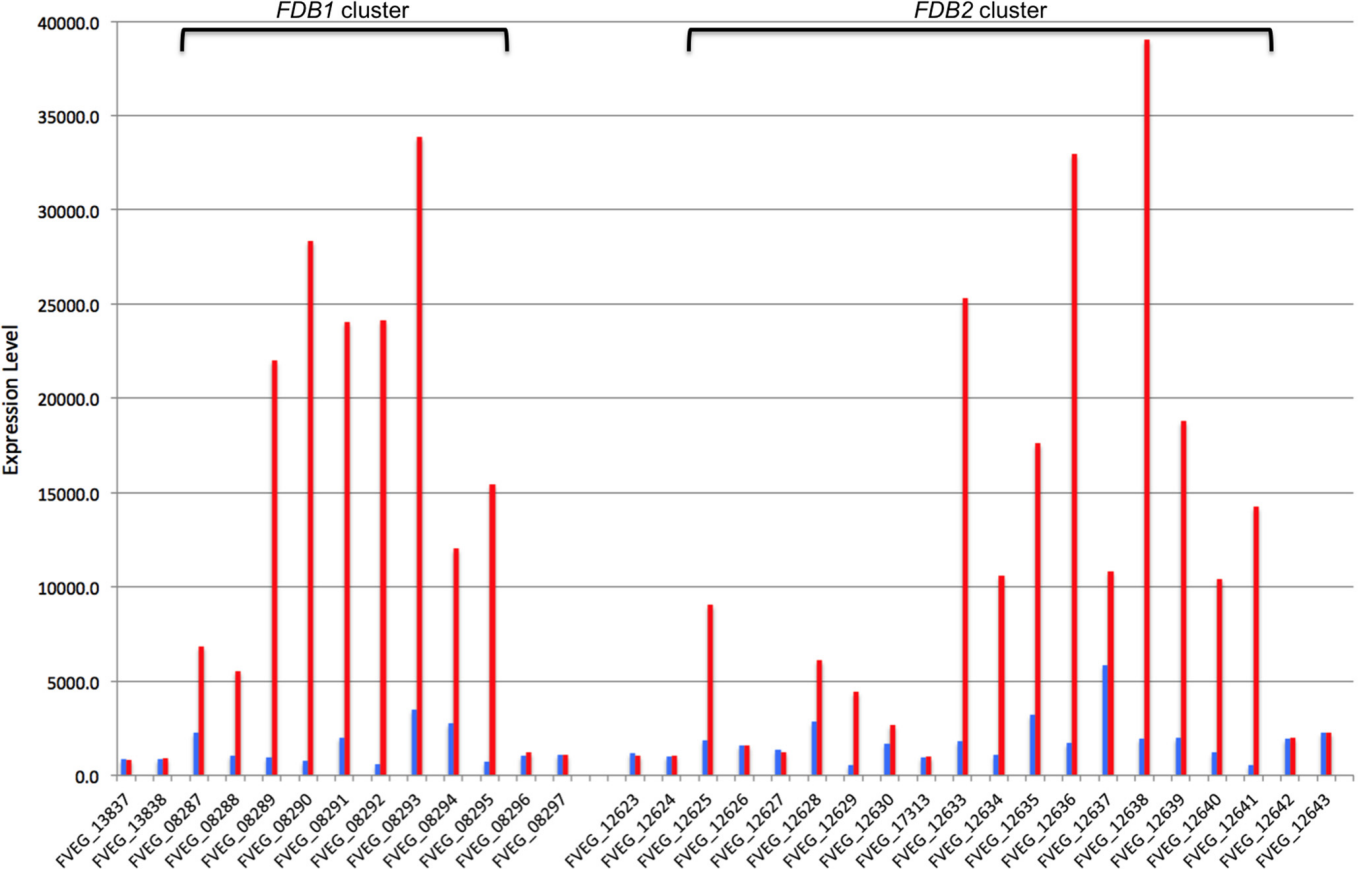
Gene expression analysis of *F*. *verticillioides* exposed to BOA. Microarray expression values for selected genes of *F*. *verticillioides* grown for two hours in PDB supplemented with either ethanol alone (blue) or BOA dissolved in ethanol (red). Gene expression values are shown for the proposed *FDB1* cluster (FVEG_08287 to FVEG_08295) and *FDB2* cluster (FVEG_12625 to FVEG_12641) as well as for genes flanking each end of the clusters. See S1 Table for more details on the genes and their fold change in expression.

Microarray analysis of wild-type strain FRC M-3125 identified 28 genes with increased expression (≥3-fold) two hours after exposure to a non-inhibitory concentration of BOA (50 μg ml^-1^). Sixteen contiguous genes (FVEG_12625 to 12641) are located along chromosome 3 and correspond to the previously described *FDB2* gene cluster in *F*. *verticillioides* [7]. Individual gene expression responses in the *FDB2* cluster ranged from no change to a 27-fold increase following exposure to BOA (Fig 2; S1 Table). Nine other genes (FVEG_08287–08295) exhibiting increased expression upon exposure to BOA (3- to 43-fold increases) were located adjacent to one another along chromosome 10 (Fig 2; Table 2; S1 Table). Their coexpression suggested they too constitute a gene cluster.

**Table 2:** Genes of the *FDB1* cluster.

FVEG Locus^a^	NCBI BLASTP Result^b^	Conserved Protein Domain Accession	E-value
08287	NmrA-like family	pfam05368	1.26e-22
08288^c^	Major facilitator superfamily transporter	pfam07690	7.94e-04
08289^c^	Amidase	pfam01425	7.45e-43
08290	Dienelactone hydrolase	pfam01738	4.84e-20
08291	Metallo-β-lactamase superfamily	pfam00753	5.62e-05
08292	Amino acid transporter	pfam01490	1.83e-17
08293	Salicylate hydroxylase	TIGR03219	1.06e-66
08294	Zn(II)2Cys6 transcription factor	pfam04082	9.89e-23
08295	Amidase	pfam01425	3.37e-97

^a^*F*. *verticillioides* locus tag within the Broad Institute *Fusarium* Comparative Database (http://www.broadinstitute.org/annotation/genome/fusarium_group/MultiHome.html).

^b^Protein sequences from the Broad database were used to assign putative functions based on BLASTp searches of the non-redundant databases (default parameters) at the National Center for Biotechnology Information (http://blast.ncbi.nlm.nih.gov) and the accompanying Conserved Domain Database. The presented E-values are based on the conserved protein domain best match.

^c^The annotations of FVEG_08288 and FVEG_08289 were collapsed into the single annotated gene FVEG_16285 in version 5 of the genome. However, data indicate this revision is in error, and we retain the previous annotation of these two separate genes.

Reciprocal BLAST searches using protein sequences indicated FVEG_08289 –FVEG_08294 are paralogs of the *FDB2* cluster genes FVEG_17313, FVEG_12625, FVEG_12637, FVEG_12640, FVEG_12630, FVEG_12635, respectively (S1 Table). Given this paralogy and the coexpression data, FVEG_08287–08295 were proposed to constitute the *FDB1* gene cluster. Five of the paralogs were induced to a much greater degree in the *FDB1* cluster compared to the *FDB2* cluster (Fig 2). The transcription factor gene FVEG_12635 in the *FDB2* cluster was the exception because it exhibited a slightly higher level of induction than its *FDB1* cluster paralog FVEG_08294. While the putative metallo-β-lactamase gene FVEG_08291 exhibited greater induction in response to BOA compared to its *FDB2* paralog FVEG_12637, the latter was expressed more than FVEG_08291 in the absence of BOA. Of all the genes in the *FDB1* and *FDB2* clusters, FVEG_12637 was the most highly expressed gene in the absence of BOA.

The *FDB1* cluster genes FVEG_08289 and FVEG_08295 both encode putative amidases (Table 2), though of different subclasses. These two genes were induced 23- and 22-fold, respectively, by exposure to BOA (S1 Table). The *FDB2* cluster gene FVEG_17313 (previously annotated as two genes, FVEG_12631 and FVEG_12632) is a paralog of FVEG_08289, whereas FVEG_08295 does not have a paralog in the *FDB2* cluster.

### Demonstration that the *FDB1* gene cluster corresponds to the *FDB1* locus, and identification of genes essential for BOA tolerance

**Fig 3:**
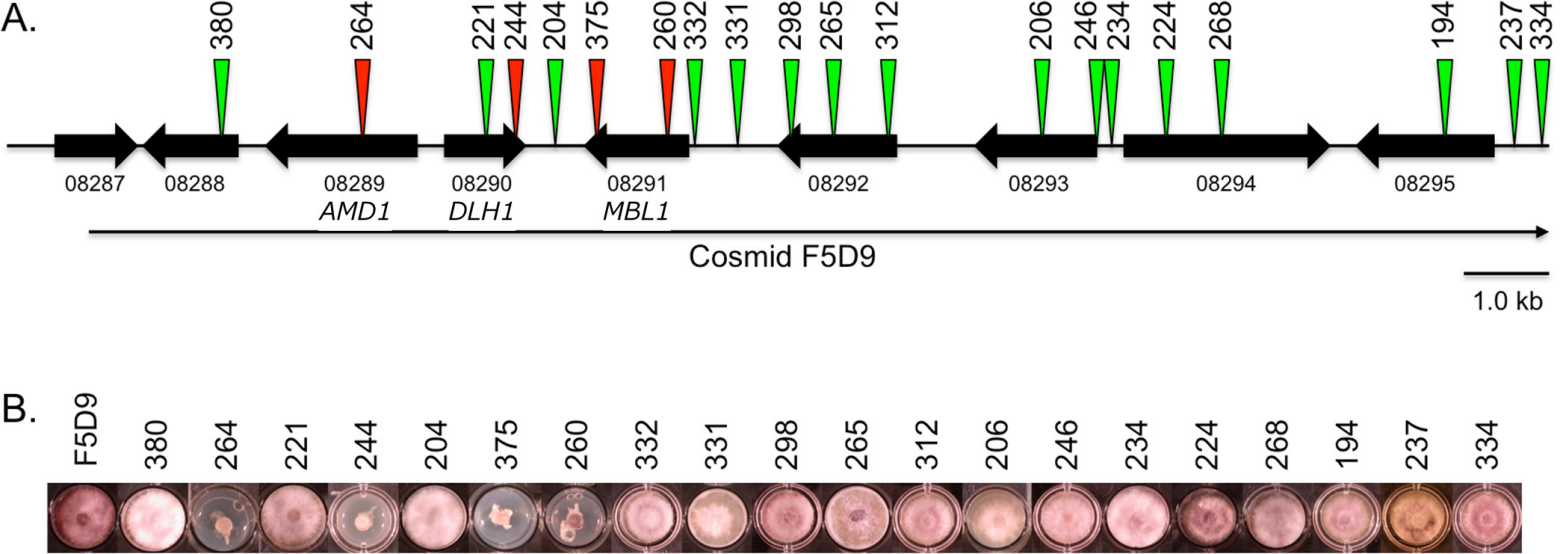
Complementation assay of the *F*. *verticillioides fdb1* mutant with clones derived from transposon mutagenesis of cosmid F5D9. (A) Chromosome 10 genes FVEG_08287 –FVEG_08295 (black block arrows) from the *FDB1* gene cluster are shown in their transcriptional orientations. Cosmid clone F5D9 (long, thin black arrow) includes full-length copies of these genes except for FVEG_08287, which is truncated. Green and red triangles indicate the positions of transposon insertions in 20 different F5D9-derived clones following mutagenesis. Green indicates transposon insertions that did not affect complementation of the *fdb1* mutant, whereas red indicates insertions that affected complementation. (B) Growth phenotypes of strain AEG 74-A4-3 transformed separately with clone F5D9 and 20 F5D9-derived clones on BOA medium (0.9 mg ml^-1^). Transformation of strain AEG 74-A4-3 with clone F5D9 and most F5D9-derived clones (green triangles) restored wild-type growth in the presence of BOA. Four F5D9-derived clones (red triangles; insertions 244, 264, 260 and 375) did not restore wild-type growth in the presence of BOA. The four latter transformants were also not able to metabolize BOA.

Cosmid clone F5D9, containing 46.6 kb of *F*. *verticillioides* genomic DNA corresponding to chromosome 10 supercontig 11:4512–51137 in the Broad Institute genome sequence database, was determined to include the entire *FDB1* cluster except for the 5’ half of gene FVEG_08287 (Fig 3). Clone F5D9 complemented the previously characterized, meiotically defined *fdb1* mutation in strain AEG 74-A4-3 [8] by restoring BOA tolerance (Fig 3), thus demonstrating the cosmid contained the functional allele of the *FDB1* locus. To address the question of which gene or genes in the *FDB1* cluster are essential for complementation, cosmid F5D9 was subjected to transposon mutagenesis to generate individual clones having a single transposon sequence inserted at a random position along F5D9. Clones were screened by sequence analysis to identify those having an insert within the *FDB1* cluster. Thirteen of the clones had an insert within the coding region of a *FDB1* cluster gene, and seven clones had an insert in an intergenic region (Fig 3). Each of the 20 F5D9-derived clones was transformed separately into strain AEG 74-A4-3 to determine whether the transposon insertion affected the ability of the clone to complement the *fdb1* mutation. Transformants carrying any one of 16 F5D9-derived insertion clones each exhibited BOA tolerance similar to that of wild-type *F*. *verticillioides*, whereas transformants carrying any one of the other four F5D9-derived clones retained BOA sensitivity similar to that of the *fdb1* mutant (Fig 3). These latter four clones had insertions in FVEG_08289 (insertion #264), FVEG_08290 (insertions #244), or FVEG_08291 (insertions #375 and #260). We have designated these three genes *AMD1*, *DLH1*, and *MBL1*, respectively, due to their predicted protein domain homologies (Table 2). *AMD1* was predicted to encode an amidase (pfam01425), *DLH1* was predicted to encode a dienelactone hydrolase (pfam01738), and *MBL1* was predicted to encode a metallo-beta-lactamase (pfam00753). The deduced Mbl1 protein sequence contained the HxHxDHxG amino acid sequence motif at residues 147–154 that is expected of the class B metallo-β-lactamase superfamily (S1 Fig). As noted above, FVEG_08291 in the *FDB1* cluster and FVEG_12637 in the *FDB2* cluster are reciprocal BLAST best matches within *F*. *verticillioides*. Therefore, we have designated FVEG_12637 as *MBL2*. Whether the *MBL2*-encoded protein, Mbl2, is functional was not determined, but Mbl1 and Mbl2 are 65% identical along their aligned region (S1 Fig). The deduced Mbl2 sequence includes the core domain without additional upstream and downstream amino acid sequences present in Mbl1.

Significant induction of *AMD1* (FVEG_08289), *DLH1* (FVEG_08290), and *MBL1* (FVEG_08291) in BOA-exposed cultures after 30 min incubation was confirmed by qPCR. Normalized expression differences relative to the untreated control were calculated based on ΔΔCt. *AMD1* induction was 19282-fold (range, 15466–24041), compared to 459-fold (range, 210–1006) for *DLH1*, and the 427-fold (range, 172–1057) for *MBL1*. These expression values generally support those from the microarray analysis even though qPCR was conducted on samples from an earlier time point (30 min versus 120 min).

**Fig 4:**
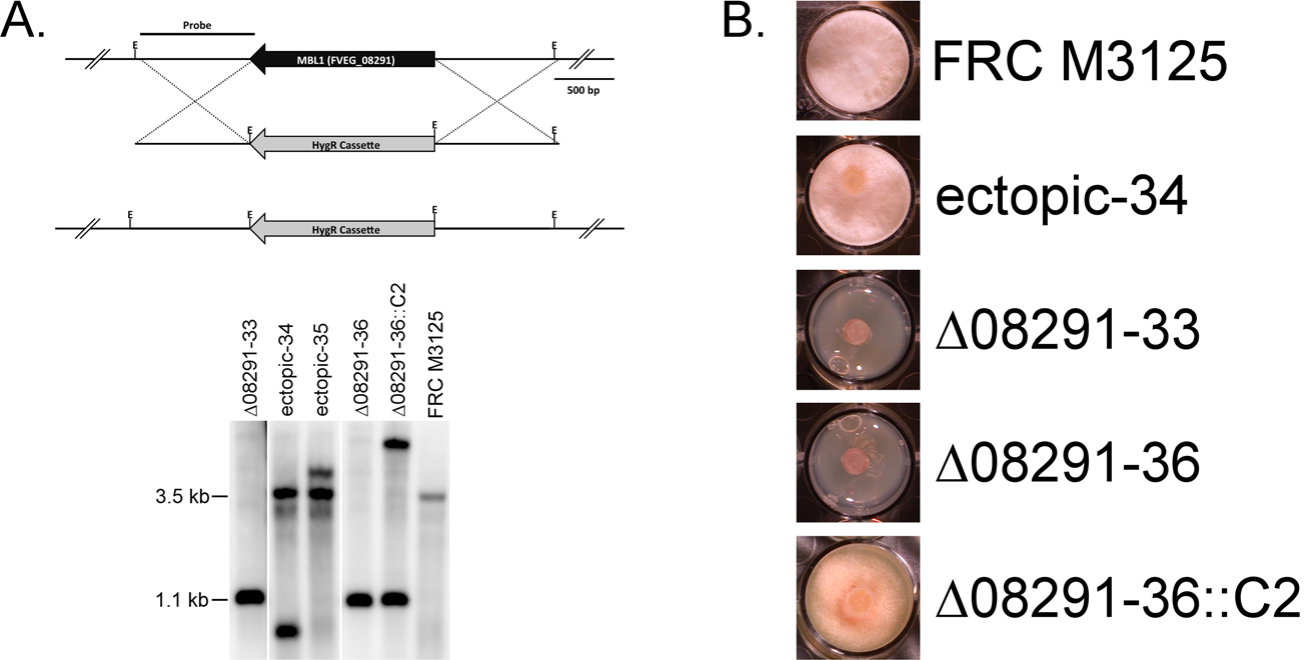
Deletion analysis of *MBL1*. (A) FVEG_08291 (*MBL1*) restriction map of the native and deletion alleles and Southern hybridization analysis of transformants showing the banding patterns of wild-type strain FRC M-3125 and the transformants having either homologous integration and deletion of the ORF or ectopic integration. Complementation of one of the deletion transformants is also shown (Δ08291–36::C2). Genomic DNA from all strains was digested with EcoRI (E). Wild type had a ~3.5 kb fragment whereas the deletion allele was 1.1 kb. The flank of the gene was used as probe as shown. The blot was cropped to show only the lanes of interest. (B) Growth phenotypes on BOA-amended agar of wild-type FRC M-3125 and *Δmbl1* mutants targeting FVEG_08291. Mutants Δ08291–33 and Δ08291–36 were unable to grow on BOA (0.9 mg ml^-1^), and add-back of the gene (Δ08291–36::C2) complemented the deletion phenotype.

**Fig 5:**
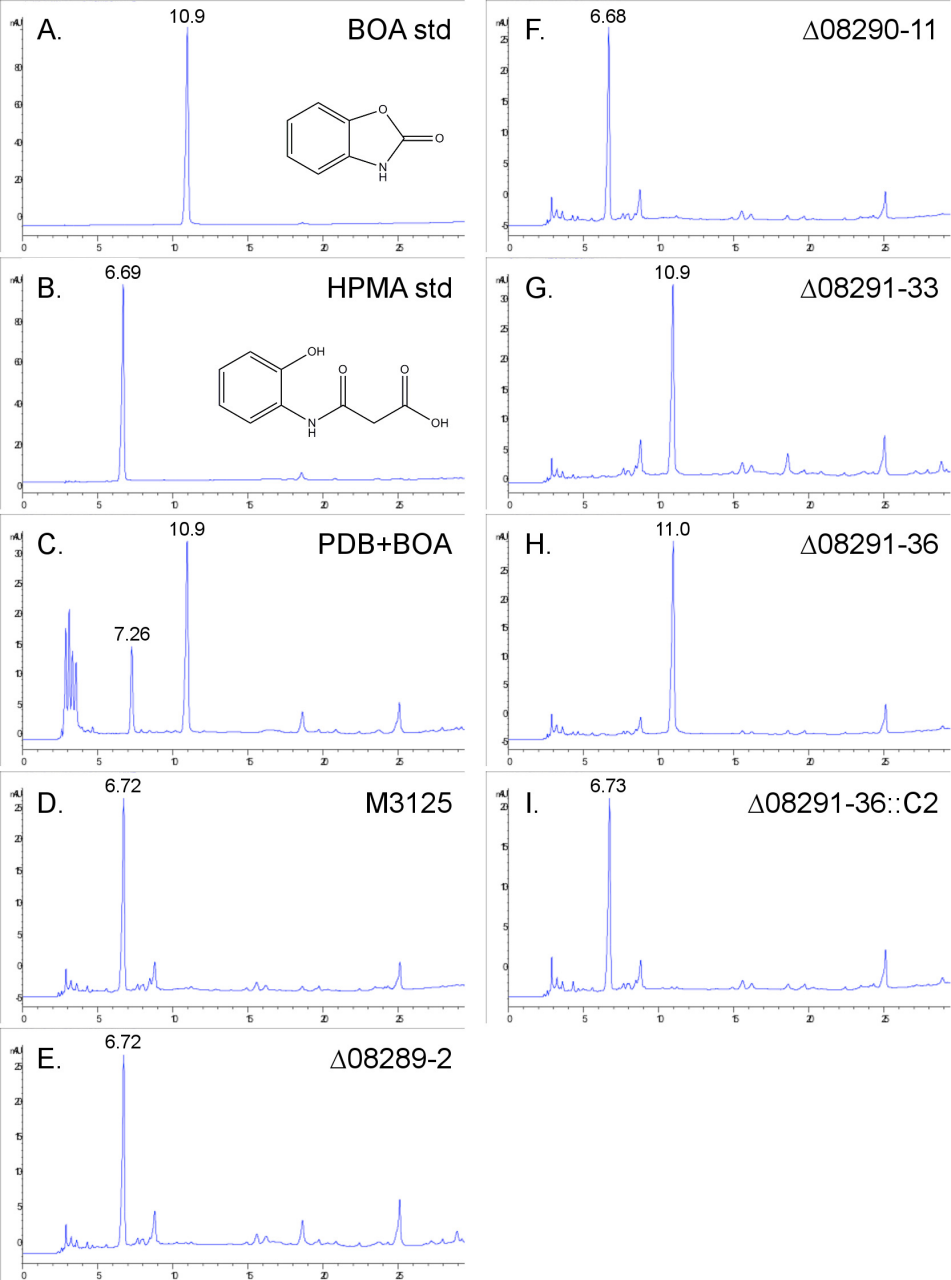
HPLC analysis of BOA detoxification into HPMA by deletion mutants. Chromatograms of (A) BOA and (B) HPMA standards (retention times of 10.9 and 6.69 min, respectively) in comparison to PDB amended with BOA that was either (C) left uninoculated (PDB+BOA) or was inoculated with (D) wild-type FRC M-3125 or the deletion mutants of (E) FVEG_08289 (*AMD1*), (F) FVEG_08290 (*DLH1*), or (G-H) FVEG_08291 (*MBL1*; two different deletion mutants). Wild type, *Δamd1*, and *Δdlh1* were all able to fully metabolize BOA to HPMA. Two *Δmbl1* mutants, Δ08291–33 and Δ08291–36, were unable to metabolize BOA. (I) Add-back of *MBL1* (Δ08291–36::C2) complemented the deletion.

*AMD1*, *DLH1*, and *MBL1* were deleted in wild-type strain FRC M-3125, but unlike the transposon mutagenesis and cosmid complementation assay, only *MBL1* (FVEG_08291) was essential for tolerance and metabolism of BOA (Figs 4 and 5). The Δ*mbl1* mutants Δ08291–33 and Δ08291–36 did not grow in the presence of BOA, which is consistent with their inability to degrade the compound. Complementation of the Δ*mbl1* mutation (Δ08291–36::C2) conferred wild-type tolerance to BOA and the ability to metabolize BOA to HPMA (Figs 4 and 5). The *Δamd1* (Δ08289–2) and *Δdlh1* (Δ08290–11) mutants had the same qualitative tolerance to BOA as the wild type and were able to fully metabolize BOA to HPMA (Fig 5; S2 and S3 Figs). In contrast to BOA, 2-oxindole was fully metabolized by the Δ*mbl1* mutants (S4 Fig).

**Fig 6:**
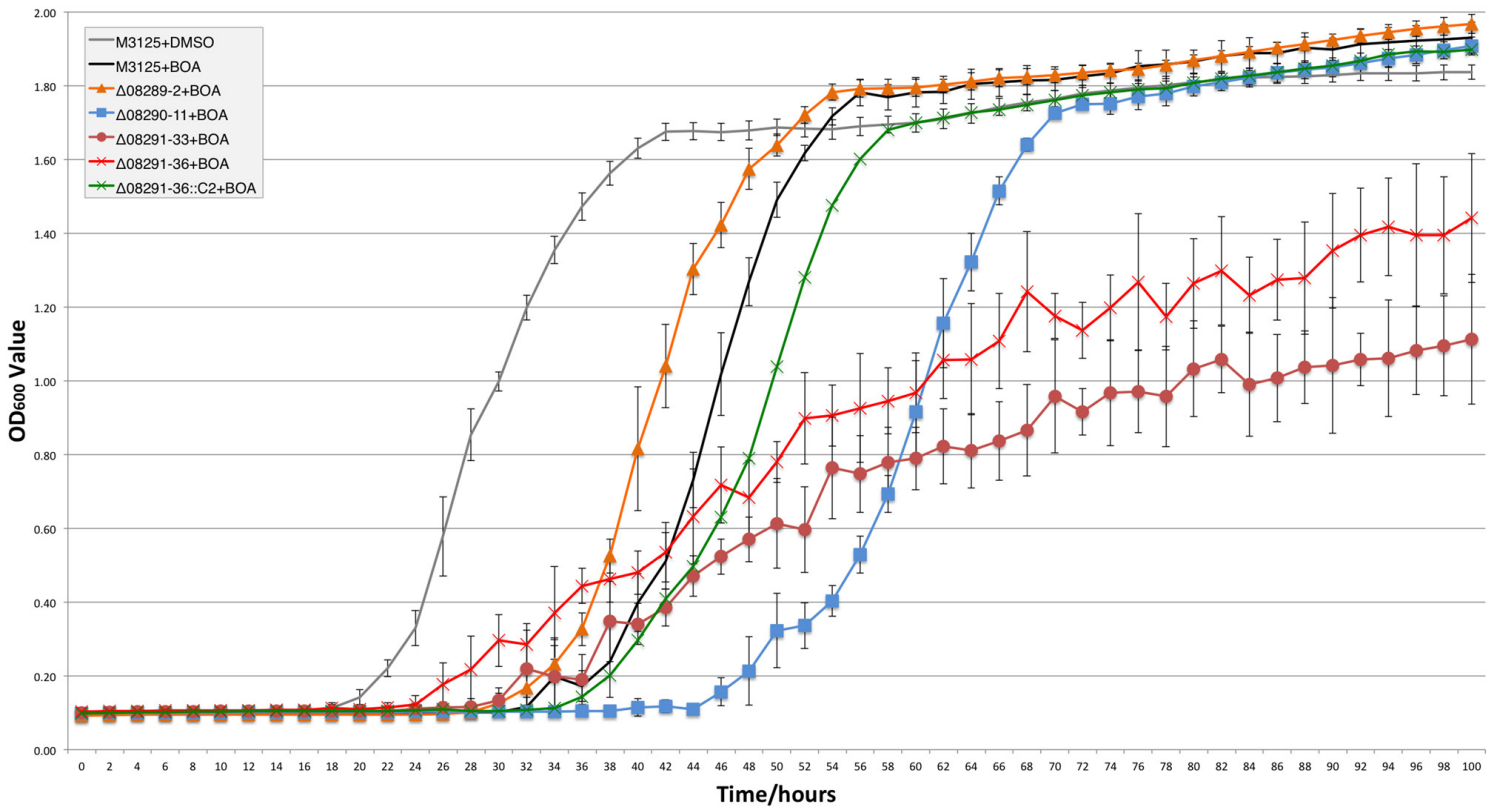
Growth curve analysis of deletion strains in comparison to wild type. Strains were monitored for 100 h in PDB amended with BOA (0.5 mg ml^-1^). OD_600_ measurements recorded every 2 h were plotted (mean ± standard deviation). For this experiment BOA was dissolved in DMSO, and a DMSO-only control for wild-type strain FRC M-3125 is shown (grey line). FRC M-3125 in PDB with BOA is shown with a black line. The *Δamd1* strain (Δ08289–2) is the orange line with triangles. The *Δdlh1* mutant (Δ08290–11) is the blue line with squares. The *Δmbl1* mutants, Δ08291–33 and Δ08291–36, are the brown line with circle markers and red line with × markers, respectively. The *Δmbl1*::*MBL1* complemented strain (Δ08291–36::C2) is the green line with × markers. Experiment was conducted twice with similar results. Data from one experiment is presented.

Evaluation of the Δ*mbl1* mutants (Δ08291–33 and Δ08291–36) in liquid PDB plus BOA (0.5 mg ml^-1^) showed that both mutants exhibited reduction in growth compared to wild-type strain FRC M-3125 (Fig 6). The complemented strain Δ08291–36::C2 grew as well as the wild type, as did *Δamd1* (Δ08289–2). The *Δdlh1* mutant (Δ08290–11) had reduced growth, showing a more subtle effect not evident from the assay using PDA amended with a higher amount of BOA (0.9 mg ml^-1^).

### Assessment of virulence and fumonisin production

All of the fungal strains (wild type and mutants) reduced the dry weight of inoculated maize seedlings by 45–57% compared to the dry weight of the uninoculated seedlings. Necrotic leaf lesions and other typical disease symptoms were observed on maize seedlings from all fungal treatments. All mutants were capable of causing seedling blight comparable to wild type.

Since the sweet corn Silver Queen used in our seedling disease assays is known to be sensitive to the phytotoxic effects of FB1 [20, 21], the fumonisin production capability of the mutants was compared to their wild-type progenitor. The individual gene deletion strains did not exhibit any consistent differences in fumonisin production. In general, all the genetically modified strains produced less FB1, FB2, and FB3 than did wild-type strain FRC M-3125. For example, FRC M-3125 produced FB1 at an average concentration of 564 μg/g on cracked maize, whereas *Δamd1* (Δ08289–2) produced 390 μg/g and *Δdlh1* (Δ08290–11) produced 241 μg/g. The two *Δmbl1* mutants, Δ08291–33 and Δ08291–36, produced widely different average FB1 concentrations of 259 and 447 μg/g, respectively. The complemented *Δmbl1* strain, Δ08291–36::C2, which is derived from Δ08291–36, produced 225 μg/g FB1. Uninoculated control replicates contained an average FB1 concentration of 0.42 μg/g.

### Species distribution and phylogenic relationships of the *FDB1* and *FDB2* gene clusters

**Fig 7:**
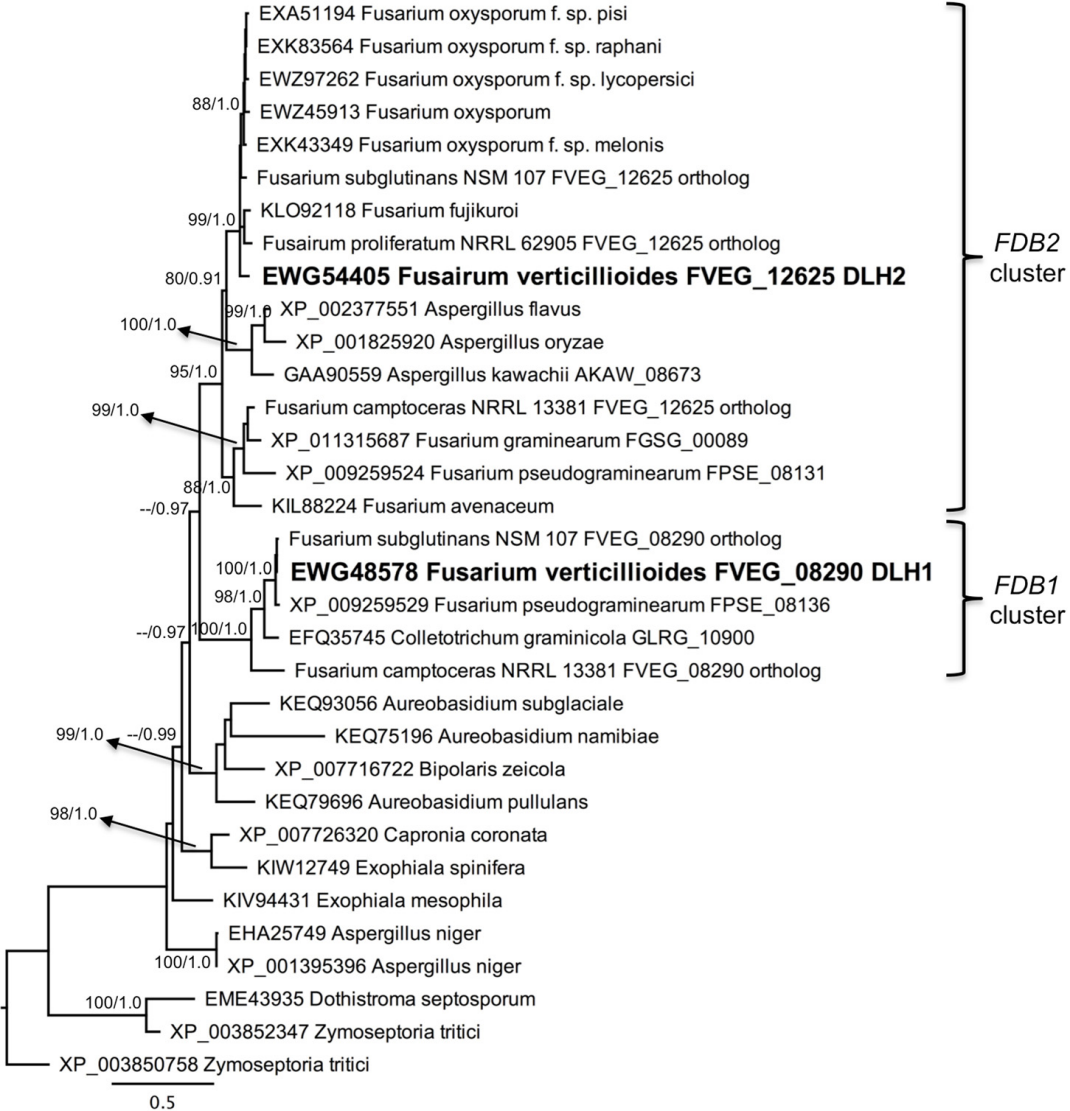
Protein based phylogeny of FVEG_08290 (*DLH1*) and FVEG_12625 (*DLH2*). The PhyML cladogram is shown with bootstrap values (200 replications) indicated for branches having ≥80% support. Bayesian posterior probabilities are also indicated for those branches. *Zymoseptoria tritici* XP_003850758 was the designated outgroup.

**Fig 8:**
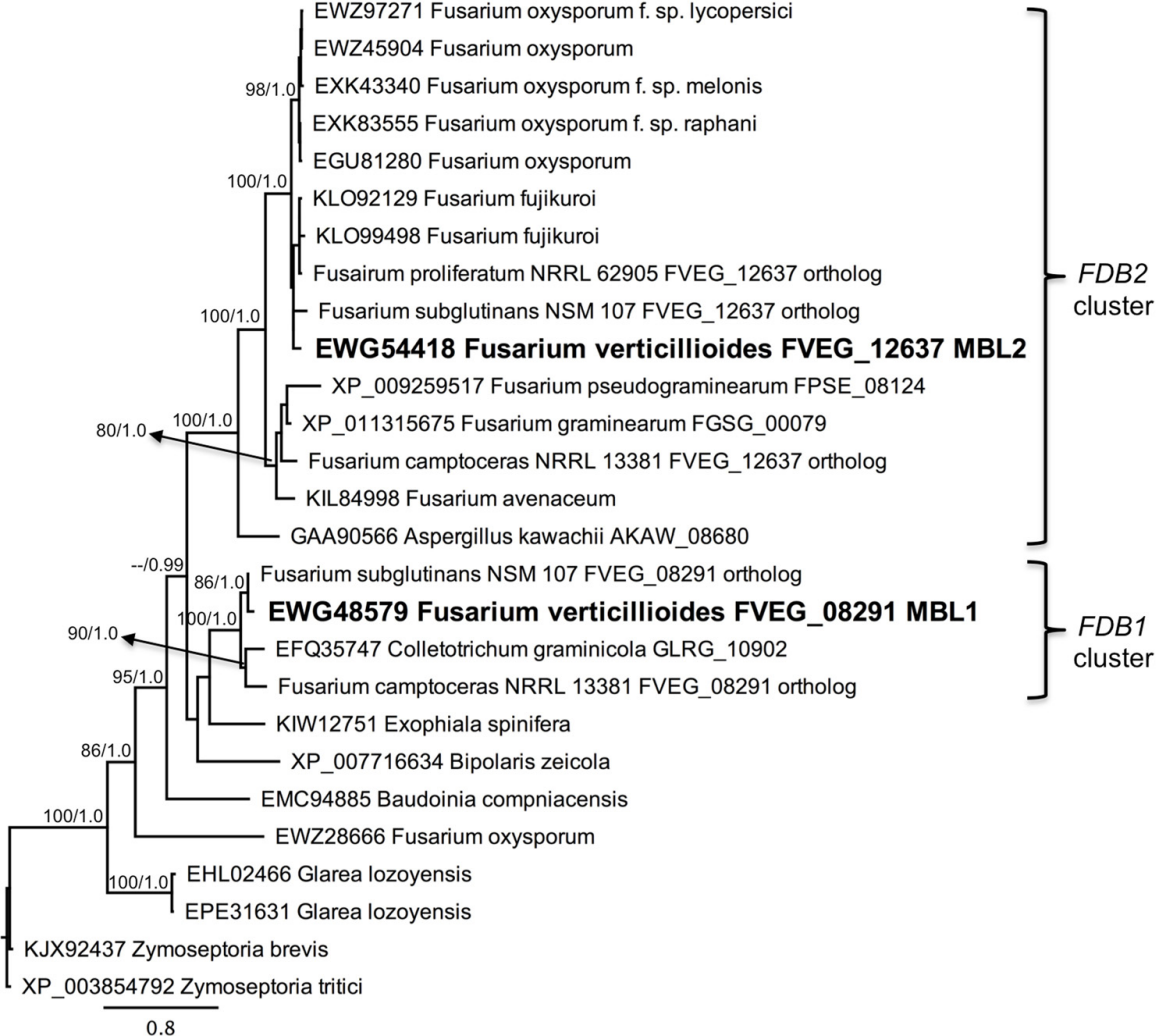
Protein based phylogeny of FVEG_08291 (*MBL1*) and FVEG_12637 (*MBL2*). The PhyML cladogram is shown with bootstrap values (200 replications) indicated for branches having ≥80% support. Bayesian posterior probabilities are also indicated for those branches. *Zymoseptoria tritici* XP_003854792 was the designated outgroup.

BLAST searches of NCBI and a custom database of *Fusarium* genome sequences were conducted using each of the genes from the *FDB1* and *FDB2* clusters. Of 49 *Fusarium* species screened, only *F*. *verticillioides*, *F*. *subglutinans*, and *Fusarium camptoceras* had the full set of *FDB1* cluster genes at a distinct locus separate from *FDB2* cluster genes (Table 3). Twenty-five species had the *FDB2* cluster but not the *FDB1* cluster. No species had the *FDB1* cluster only. Species were considered to have the *FDB2* cluster if they had homologs of all or a majority of the *F*. *verticillioides FDB2* genes, including the signature *NAT1* gene [7, 12, 13]. Nine species had only a portion of the *FDB2* cluster that did not include *NAT1*, and an additional 12 species lacked both *FDB* clusters. Of particular note was the wheat pathogen *F*. *pseudograminearum*, because while it did not have a *FDB1* gene cluster distinct from its *FDB2* cluster (Table 3), it did have orthologs of *DLH1* and *AMD1* (Fig 7; S5 Fig) located adjacent to its previously delineated *FDB2* cluster [10, 23]. Thus, an extended view of the *F*. *pseudograminearum FDB2* cluster would include its *DLH1* (FPSE_08136) and *AMD1* (FPSE_08135) homologs in addition to the *DLH2* (FPSE_08131) homolog (Fig 7; S5 Fig), all located downstream of the *NAT1* (FPSE_08123) homolog [10, 35]. *Fusarium pseudograminearum* does not possess an ortholog of *F*. *verticillioides AMD2* (S5 Fig), nor does it possess a *MBL1* ortholog (Fig 8). Instead, the *F*. *pseudograminearum* γ-lactamase gene recently described by Kettle et al. [23], which they designated as *FDB1*, is an ortholog of the *F*. *verticillioides* gene *MBL2* from the *FDB2* cluster (Fig 8). Since *F*. *verticillioides*, *F*. *subglutinans*, and *F*. *camptoceras* are the only species known to have the *FDB1* cluster, they are likewise the only species currently known to have the *MBL1* gene.

**Table 3:** *Fusarium* genomes assessed by BLASTn for presence of the *FDB1* and *FDB2* clusters.

				Cluster Present^c^	
*Fusarium* Species	Strain	Species Complex	Source of Data^a^	*FDB1*	*FDB2*
*F*. *acuminatum*	CS5907	*tricinctum*	NCBI	No	No
*F*. *anthophilum*	NRRL 25214	*fujikuroi*	NCAUR	No	Yes
*F*. *armeniacum*	FRC R-09335	*sambucinum*	NCAUR-MS^b^	No	Yes
*F*. *avenaceum*	Fa05001	*tricinctum*	NCBI	No	Partial
*F*. *aywerte*	NRRL 25410	*chlamydosporum*	NCAUR-IT	No	No
*F*. *babinda*	NRRL 25539	*babinda*	NCAUR-IT	No	No
*F*. *bulbicola*	NRRL 22947	*fujikuroi*	NCAUR	No	Yes
*F*. *camptoceras*	NRRL 13381	*incarnatum*-*equiseti*	NCAUR-MS	Yes	Yes
*F*. *circinatum*	FSP 34	*fujikuroi*	NCBI	No	Yes
*F*. *commune*	NRRL 28387	*nisikadoi*	NCAUR-IT	No	Partial
*F*. *culmorum*	CS7071	*sambucinum*	NCBI	No	Yes
*F*. *dlaminii*	NRRL 13164	*fujikuroi*	NCAUR & BGI	No	No
*F*. *foetens*	NRRL 38302	*oxysporum*	NCAUR-IT	No	Yes
*F*. *fujikuroi*	B14	*fujikuroi*	NCAUR-MS	No	Yes
*F*. *gaditjirrii*	NRRL 45417	*nisikadoi*	NCAUR-IT	No	No
*F*. *globosum*	NRRL 26131	*fujikuroi*	NCAUR-MS	No	Yes
*F*. *graminearum*	PH-1	*sambucinum*	Broad, MIPS	No	Yes
*F*. *guttiforme*	NRRL 22945	*fujikuroi*	NCAUR-MS	No	Partial
*F*. *kyushuense*	NRRL 25348	*sambucinum*	NCAUR-MS	No	Yes
*F*. *longipes*	NRRL 20695	*sambucinum*	NCAUR-MS	No	No
*F*. *miscanthi*	NRRL 26231	*nisikadoi*	NCAUR-IT	No	Yes
*F*. *napiforme*	NRRL 25196	*fujikuroi*	NCAUR-MS	No	Partial
*F*. *oxysporum*	Fol4287	*oxysporum*	Broad	No	Yes
*F*. *phyllophilum*	NRRL 13617	*fujikuroi*	NCAUR-MS	No	Partial
*F*. *poae*	NRRL 26941	*sambucinum*	NCAUR-IT, MS	No	No
*F*. *proliferatum*	NRRL 62905	*fujikuroi*	NCAUR & BGI	No	Yes
*F*. *pseudograminearum*	CS3096	*sambucinum*	NCBI	No	Yes
*F*. *redolens*	NRRL 22901	*redolens*	NCAUR-IT	No	Yes
*F*. *sacchari*	FRC M-6865	*fujikuroi*	NCAUR & BGI	No	Yes
*F*. *sambucinum*	FRC R-6380	*sambucinum*	NCAUR-MS	No	No
*F*. *scirpi*	FRC R-06979	*incarnatum*-*equiseti*	NCAUR-MS	No	Yes
*F*. *solani f*. *sp*. *pisi*	77-13-4	*solani*	JGI	No	No
*F*. *sporotrichioides*	NRRL 3299	*sambucinum*	NCAUR-MS	No	No
*F*. *subglutinans*	NSM 107	*fujikuroi*	NCAUR & BGI	Yes	Yes
*F*. *succisae*	NRRL 13298	*fujikuroi*	NCAUR & BGI	No	Yes
*F*. *thapsinum*	FRC M-6563	*fujikuroi*	NCAUR & BGI	No	Partial
*F*. *torulosum*	ITEM 843	*tricinctum*	NCAUR-MS	No	Partial
*F*. *udum*	NRRL 25194	*fujikuroi*	NCAUR-MS	No	Partial
*F*. *verticillioides*	FRC M-3125	*fujikuroi*	Broad, MIPS	Yes	Yes
*Fusarium* sp.	NRRL 13444	*chlamydosporum*	NCAUR-MS	No	Partial
*Fusarium* sp.	Fv2	*fujikuroi*	NCAUR-MS	No	Yes
*Fusarium* sp.	NRRL 43304	*fujikuroi*	NCAUR-MS	No	Yes
*Fusarium* sp.	NRRL 52700	*fujikuroi*	NCAUR & BGI	No	No
*Fusarium* sp.	ITEM 11294	*incarnatum*-*equiseti*	NCAUR-MS	No	Yes
*Fusarium* sp.	ITEM 11363	*incarnatum*-*equiseti*	NCAUR-MS	No	Yes
*Fusarium* sp.	ITEM 7155	*incarnatum*-*equiseti*	NCAUR-MS	No	Yes
*Fusarium* sp.	ITEM 11348	*incarnatum*-*equiseti*	NCAUR-MS	No	No
*Fusarium* sp.	NRRL 36351	*sambucinum*	NCAUR-MS	No	Yes
*Fusarium* sp.	NRRL 25184	undescribed	NCAUR-IT	No	Yes

^a^NCBI = National Center for Biotechnology Information. NCAUR = U.S. Department of Agriculture, Agricultural Research Service, National Center for Agricultural Utilization Research. BGI = Beijing Genome Institute-Hong Kong. Broad = Broad Institute. MIPS = Munich Information Center for Protein Sequences. JGI = U.S. Department of Energy, Joint Genome Institute.

^b^NCAUR-MS = sequenced at NCAUR using the Illumina MiSeq platform. NCAUR-IT = sequenced at NCAUR using the Ion Torrent platform.

^c^The *FDB1* and *FDB2* clusters were noted as being present (yes) or absent (no) from the respective genomes. Each cluster was considered present if most of the associated genes were evident and contiguous. For those species having both clusters, the *FDB1* cluster was not linked to the *FDB2* cluster. Additionally for *FDB2*, the cluster had to contain an FVEG_12636 (*NAT1*) ortholog to be considered a complete cluster. Partial clusters lacked this gene but possessed other *FDB2* genes.

**Fig 9:**
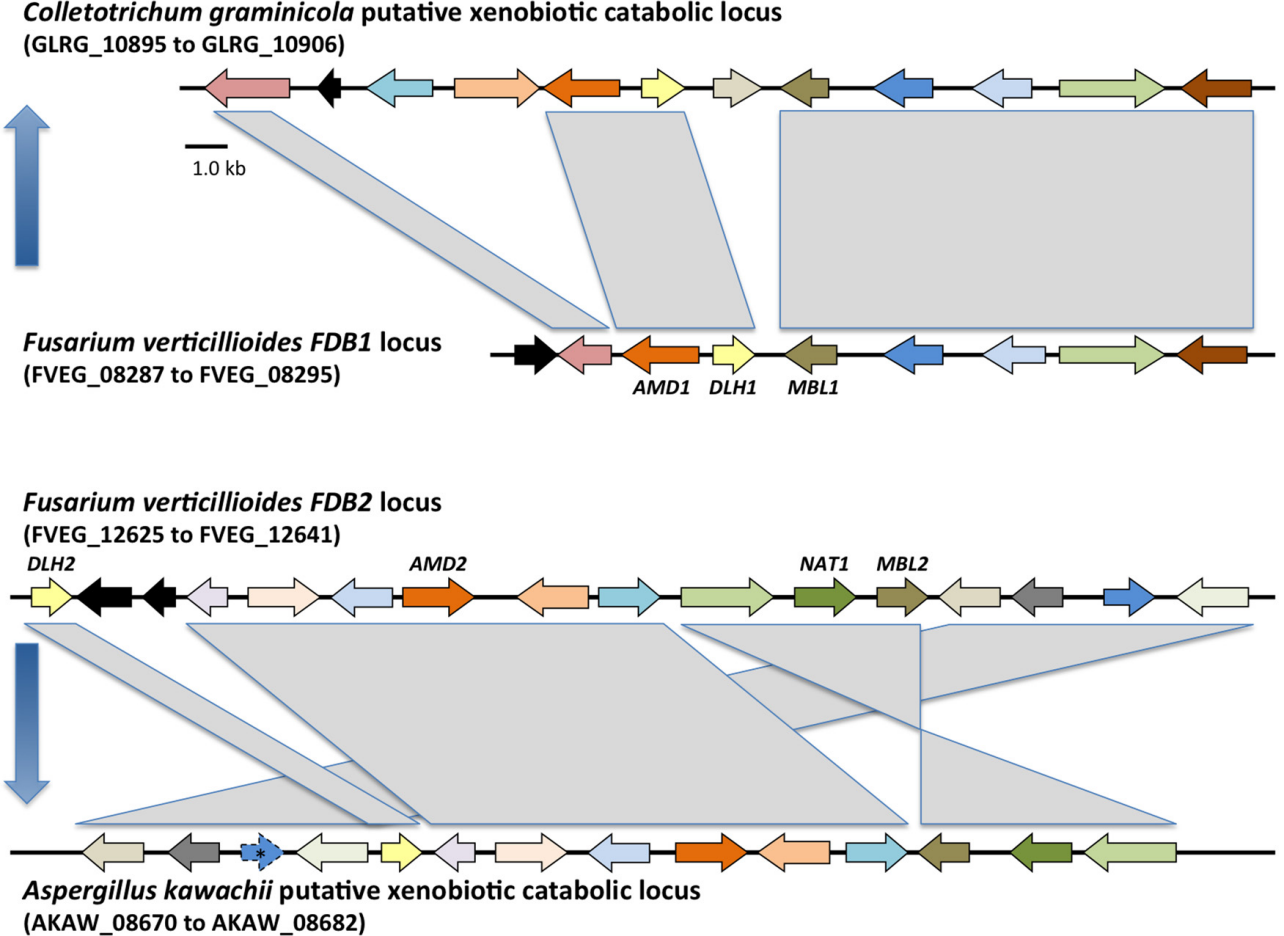
Conserved synteny of the *FDB1* cluster homologs in *F*. *verticillioides* and *C*. *graminicola*, and the *FDB2* cluster homologs in *F*. *verticillioides* and *A*. *kawachii*. The genes are color coded to show orthologs/paralogs across the clusters. As the primary genes discussed in this paper, *AMD1*/*AMD2*, *DLH1*/*DLH2*, *MBL1*/*MBL2*, and *NAT1* are noted. The blue arrows depict the predicted horizontal transfers of the *FDB1* cluster from *Fusarium* to *Colletotrichum* and the *FDB2* cluster from *Fusarium* to *Aspergillus*. Based on the synteny comparisons, an unannotated potential pseudogene for an amino acid transporter is noted with an asterisk in the *A*. *kawachii* cluster.

Individual phylograms were generated from protein sequence alignments for six of the genes in the *F*. *verticillioides FDB1* cluster having paralogs in the *FDB2* cluster. Representative phylograms are presented in Figs 7 and 8 and in S5 and S6 Figs. Interestingly, *Colletotrichum graminicola* has an intact *FDB1* gene cluster, and the phylogenies consistently resolved *FDB1* cluster protein sequences from this fungus in a clade with sequences from *F*. *camptoceras*, *F*. *verticillioides*, and *F*. *subglutinans*, which are the only species with complete *FDB1* and *FDB2* clusters. To date, *C*. *graminicola* is the only species known to possess an *FDB1* cluster but no *FDB2* cluster. A second observation from the phylogenies was that the more broadly distributed *Fusarium FDB2* gene cluster was also present in *Aspergillus kawachii*, which is the only species of this genus known to have the complete *FDB2* cluster based on genome database searches (Fig 9). Other *Aspergillus* species have lost one or more of the genes (Figs 7 and 8; S5 and S6 Figs). For example *Aspergillus flavus* has a *DLH2* ortholog but does not have *MBL2* (Figs 7 and 8).

Phylogenetic topological congruence tests were supportive of HGT as an explanation for the observed species distribution of the *FBD1* and *FDB2* gene clusters. In all comparisons, topologies of the TEF phylogeny (species tree) were significantly different (*p* > 0.001) from the individual protein phylogenies derived from genes within the *FDB1* and *FDB2* clusters. We propose that two independent HGT events have occurred during the evolution of the *FDB* clusters, with the *FDB1* cluster in *C*. *graminicola* and the *FDB2* cluster in *A*. *kawachii* both being acquired from *Fusarium* species (Figs 7 and 8; S5 and S6 Figs).

The structural synteny between the *F*. *verticillioides* and *C*. *graminicola FDB1* clusters is highly conserved, as is the synteny between the *FDB2* clusters in *F*. *verticillioides* and *A*. *kawachii* (Fig 9; S7 Fig). An incongruity in the synteny includes three genes in the *C*. *graminicola FDB1* cluster that are lacking in the *F*. *verticillioides FDB1* cluster, yet homologs of these genes are present in the *F*. *verticillioides* and *A*. *kawachii FDB2* clusters. In the Broad Institute’s *Colletotrichum* Database, these three *C*. *graminicola* genes are designated GLRG_10897, GLRG_10898, and GLRG_10901 and are predicted to encode a carboxylesterase, malonyl-CoA synthetase, and aldo/keto reductase, respectively. Phylogenies of the three proteins fit the overall pattern outlined above with the *C*. *graminicola* ortholog being basally positioned relative to the *FDB2* cluster orthologs. The carboxylesterase phylogeny is presented as a representative (S6 Fig).

The origin of the two *FDB* clusters is likely due to an ancient duplication event in *Fusarium*. If the *Fusarium FDB2* cluster was derived from a duplication of the *FDB1* cluster, and if this ancestral *FDB1* cluster was transferred from *Fusarium* to *Colletotrichum*, then the lack of orthologs for the carboxylesterase, malonyl-CoA synthetase, and aldo/keto reductase in the *F*. *verticillioides FDB1* cluster could be due to deletion of the genes at some point during the evolution of this species. This conclusion suggests the *C*. *graminicola* cluster may be more representative of the progenitor *FDB1* cluster. This is further supported by the presence of homologs of the *C*. *graminicola FDB1* carboxylesterase (GLRG_10897) and aldo/keto reductase (GLRG_10901) genes in the *F*. *camptoceras FDB1* cluster (S6 and S7 Figs).

### Detoxification of BOA and production of HPMA by *F*. *camptoceras* and other *Fusarium* species

The tolerance of species to BOA and their ability to metabolize it to HPMA were compared for isolates of *F*. *camptoceras*, *F*. *graminearum*, *F*. *oxysporum*, *F*. *subglutinans*, and *F*. *verticillioides*. *Fusarium subglutinans* and *F*. *verticillioides* were most similar, both effectively producing HPMA from BOA (S8 Fig). The other three species exhibited tolerance to BOA but were not as proficient at metabolizing BOA to HPMA.

## Discussion

Phytopathogenic fungi on agricultural hosts or in field soils encounter a range of xenobiotic compounds that must be tolerated by various mechanisms, including metabolic degradation or structural alteration. *Fusarium verticillioides* is very effective at metabolizing the maize protective Bx compounds to produce the nontoxic malonamic acids HMPMA and HPMA [5, 8]. Previously we genetically identified two loci, *FDB1* and *FDB2*, that are essential for detoxification [8]. Such metabolic activity enhances the ecological fitness of *F*. *verticillioides*, which is often present as a maize endophyte at higher frequency and abundance than other fungi that are not able to metabolize the Bx xenobiotics [15]. Production of these compounds may filter out intolerant fungi, thus favoring infection by *F*. *verticillioides* and other tolerant species [6, 15, 36].

The *FDB2* locus of *F*. *verticillioides* has been functionally characterized by gene disruption and deletion, and *NAT1*, encoding a member of the arylamine *N*-acetyltransferase family, was identified as the key gene for production of the malonamic acids HMPMA and HPMA [7]. Here we extend our understanding of *F*. *verticillioides* by characterizing the *FDB1* locus, with particular focus on three genes encoding an amidase (*AMD1*), dienlactone hydrolase (*DLH1*), and metallo-β-lactamase (*MBL1*). Of the three *F*. *verticillioides* genes targeted for deletion in this study, only the *Δmbl1* mutant was unable to hydrolyze BOA and thus could not grow in its presence.

**Fig 10:**
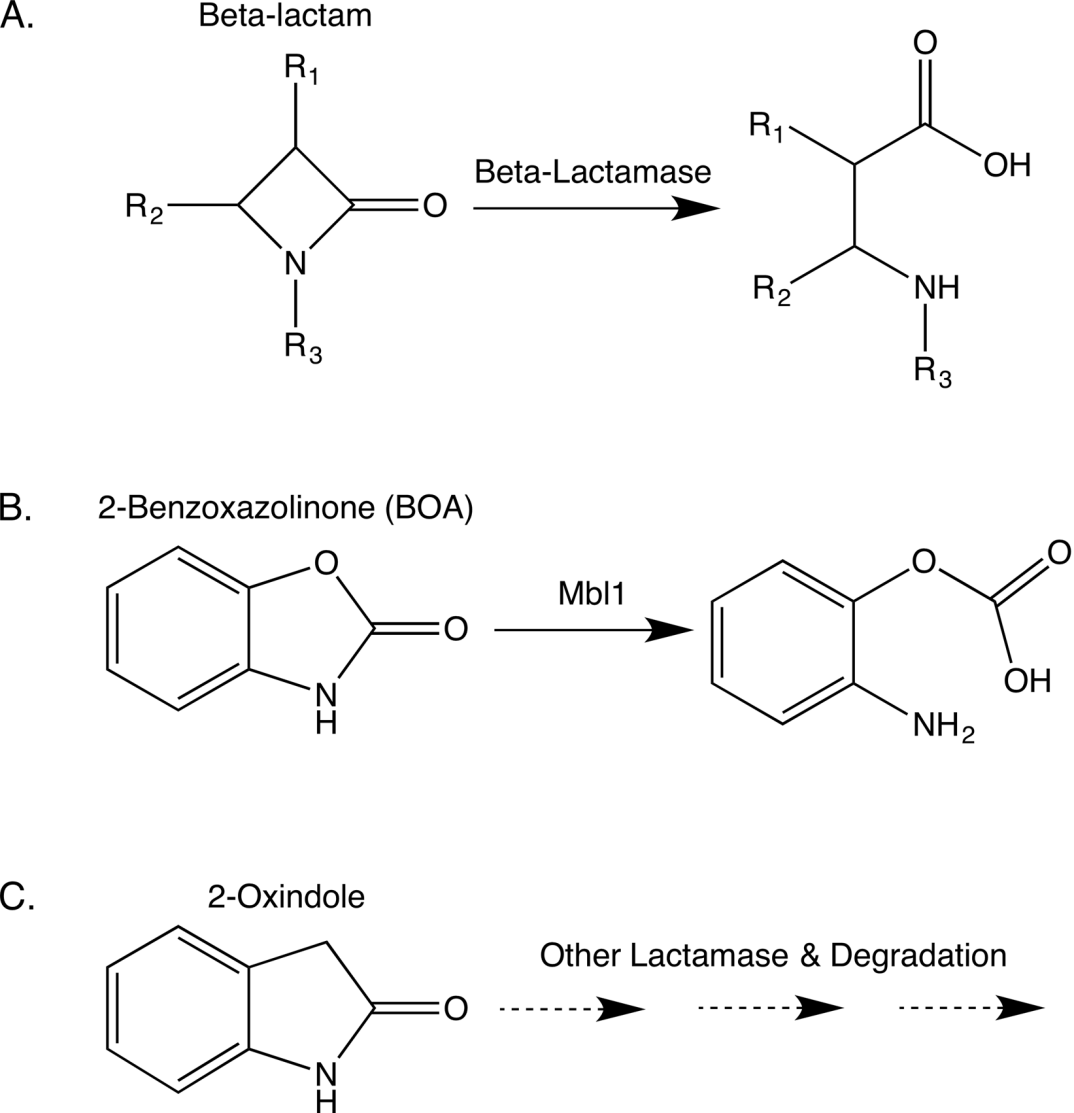
Hydrolysis and degradation of lactam xenobiotics. (A) General representation of the hydrolytic cleavage of a lactam moiety, in this case a four-atom β-lactam ring. (B) The hydrolysis of BOA by Mbl1 presumably involves a similar cleavage of the lactam moiety, resulting in production of an intermediate that is further processed to 2-aminophenol presumably by Dlh1 and/or Dlh2 encoded by the *FDB1* and *FDB2* gene cluster, respectively. (C) The lactam-containing 2-oxindole was metabolized by all strains in this study, including the *Δmbl1* mutants, thus indicating Mbl1 is not needed for the hydrolysis and degradation of this compound.

Interestingly, the predicted functions of genes in the *F*. *verticillioides FDB2* cluster suggest that the cluster encodes the necessary activities to confer tolerance to BOA by metabolizing it to HPMA. That is, the *FDB2* cluster encodes both a metallo-β-lactamase, Mbl2, with the potential to hydrolyze BOA to 2AP, and the arylamine *N*-malonyltransferase, Nat1, that catalyzes the malonylation of 2AP to form HPMA. However, several lines of evidence indicate the *F*. *verticillioides* Mbl2 does not catalyze the hydrolysis of BOA. As already noted, the *Δmbl1* mutants were unable to metabolize BOA, and additionally, the *MBL2* gene exhibited only a moderate increase in expression after exposure to BOA, whereas *MBL1* was induced 13-fold. Further, strain AEG 74-A4-3 lacks a functional *FDB1* locus but has a functional *FDB2* locus, and this strain is BOA-sensitive. Lastly, we previously showed that disruption of *MBL2* did not affect tolerance to BOA, indicating the gene is not necessary for detoxification [7]. Thus, the functionality of *MBL2* remains to be determined, and it may have distinct substrate specificities for lactam containing compounds other than BOA. We observed that 2-oxindole was fully metabolized by wild type and the *Δamd1*, *Δdlh1*, and *Δmbl1* deletion mutants, thus indicating *MBL1* is not needed for hydrolysis of 2-oxindole. This γ-lactam is structurally very similar to BOA but has a pyrrole ring rather than an oxazole ring. Whether Mbl2 or another encoded lactamase is responsible for the hydrolysis of 2-oxindole and other lactam compounds requires additional investigation (Fig 10).

Bacterial β-lactamases are well known for their hydrolysis and inactivation of β-lactam antibiotics such as penicillin. Beyond the clinical environment, β-lactamases are abundant in soil, and are produced by diverse bacterial species [37, 38]. This “soil resistome” is considered to be a reservoir of novel and potentially dangerous antibiotic resistance genes that could be transferred from the environment to bacteria of clinical importance [37]. Fungi such as *F*. *verticillioides* may be components of this soil resistome since they too undergo complex interactions with plant hosts and competing microorganisms in the plant and soil. Lactam antibiotics may mediate such microbial interactions, and they may do so without overt antibiosis. Antibiotics at concentrations below minimum inhibitory levels were shown to modulate bacterial gene expression, and consequently this has led to the proposal that antibiotics evolved not as weapons but as signaling molecules [39]. If so, lactamases in the environment may disrupt such signaling, similar to how quorum-quenching lactonases hydrolyze the *N*-acyl-L-homoserine lactone (AHL) signaling molecules ubiquitous among Gram-negative bacteria [40]. Interestingly, AHL lactonases are also members of the metallo-β-lactamase superfamily. Of the several β-lactamase domain classification subtypes known in bacteria, fungal lactamases have strong homology to mainly two subtypes, PFAM00753 (class B metallo-β-lactamases) and PFAM00144 (class C β-lactamases). Fungi may be using these enzymes to modulate bacterial signaling and gene expression (offensive strategy) as well as inactivate xenobiotics originating from plants or microbes (defensive strategy). The offensive strategy assumes secretion and extracellular activity of the fungal enzymes. The defensive strategy may involve hydrolysis of a range of β-, γ-, and higher order lactams.

The minimal information we currently know about the functions of fungal lactamases comes from two examples. The first is EncB, a metallo-β-lactamase type thioesterase produced by *Aspergillus fumigatus*. EncB does not function to hydrolyze a lactam but is involved in the biosynthesis of endocrocin, a polyketide-based secondary metabolite [41]. The second is our work detailed herein on *F*. *verticillioides MBL1* that is congruent with the results of Kettle et al. [23] characterizing the hydrolytic activity encoded by *MBL2* orthologs in *F*. *pseudograminearum* and *F*. *graminearum* against MBOA and BOA. The EncB, Mbl1, and Mbl2 examples suggest that rather than being considered traditional β-lactamases that act on four atom rings, these fungal enzymes are potentially functional on a broad range of lactams with varying ring sizes or may combine with other functional domains to act on other substrates.

Functional redundancy regarding *AMD1* and *DLH1* and their paralogs likely explains the different results observed between the cosmid complementation assay and the targeted deletions of these two genes. Strain AEG 74-A4-3 used for cosmid complementation was derived from a series of crosses involving wild-type *F*. *verticillioides* and a BOA-sensitive strain originally isolated from banana [8]. Given the uncertain genomic nature of AEG 74-A4-3, this strain may lack necessary gene expression or functionality of encoded proteins to compensate for the loss of Amd1 or Dlh1 function due to the transposon insertions in the encoding genes on cosmid clone F5D9. In contrast, targeted deletion of these two genes in wild-type strain FRC M-3125 should not interfere with the expression and function encoded by other genes. Thus, another amidase and dienelactone hydrolase likely compensated for the *Δamd1* and *Δdlh1* deletions, respectively, since these mutants retain BOA-tolerance and metabolic degradation capability. The paralog to *AMD1* in the *FDB2* cluster, FVEG_17313 (designated *AMD2*), had minimal expression in the microarray analysis and showed no induction upon BOA exposure, so it may not be the source of redundancy. In contrast, FVEG_12625 (designated *DLH2*) is the *FDB2* cluster paralog of *DLH1*, and it was distinctly induced by BOA-exposure, and thus may provide redundant function for hydrolyzing the lactone moiety in the BOA molecule. We plan to create a double deletion mutant for both *DLH1* and *DLH2* to test this potential redundancy.

*AMD1* and *DLH1* were previously noted from *F*. *pseudograminearum* as a likely two-gene cluster also present in *F*. *verticillioides* and *C*. *graminicola* in the same divergently transcribed arrangement [35]. The authors noted the potential for an ancient relationship of these and surrounding genes across these three species. Here we extend that observation with a thorough evolutionary analysis of the *FDB1* and *FDB2* gene clusters. We propose that the *FDB2* cluster arose as a duplication of the *FDB1* cluster with rearrangement and expansion by incorporating additional genes (Fig 9). The phylogenetic analyses are supportive of this duplication occurring within a *Fusarium* progenitor. The last common ancestor of *F*. *camptoceras*, *F*. *verticillioides* and other *Fusarium* species we analyzed would be at the base of the *Gibberella* clade [**42**]. Such early expansion of the *FDB* genes would mean most descendent *Fusarium* species have lost the *FDB1* cluster. Based on currently available genomic sequence data, only *F*. *camptoceras*, *F*. *verticillioides*, and *F*. *subglutinans* possess a complete *FDB1* cluster. *Fusarium pseudograminearum* possesses some of the *FDB1* genes directly linked to its *FDB2* cluster [10, 35].

Both *AMD1* and *DLH1* from *F*. *pseudograminearum* were shown to have higher expression in root and leaf tissues from wheat than from barley [35]. Deletion of *DLH1* in this fungus resulted in mutants with significantly reduced virulence toward both wheat and barley when assessed for development of root rot and crown rot, yet the mutants were not altered in their BOA-tolerance and grew as well as wild type [35]. The *Δdlh1* mutants we created in *F*. *verticillioides* were still virulent against maize seedlings in our assay, and the mutant was still able to grow on BOA as noted above, though the growth rate was reduced when monitored in liquid culture, again indicative of potential Dlh1 and Dlh2 functional redundancy.

In addition to our hypothesis that *FDB1* and *FDB2* clusters in *Fusarium* species likely arose by an ancient duplication, we also postulate that each cluster has its own history of HGT. First is the acquisition of the *FDB1* cluster by *Colletotrichum*. Since currently available genome sequences of other *Colletotrichum* species do not possess the *FDB1* cluster, it is possible that the HGT event was from a *Fusarium* progenitor directly to the species *C*. *graminicola*. Another possibility is multiple *Colletotrichum* species once possessed the *FDB1* cluster, but they have since lost it. The second proposed HGT event was transfer of the *FDB2* cluster from *Fusarium* to an *Aspergillus*, with subsequent loss of the cluster from all currently available sequenced *Aspergillus* species except *A*. *kawachii*. Species such as *A*. *parasiticus* and *A*. *flavus* possess some of the *FDB2* genes but not all, supporting the conclusion that the intact cluster was not retained. Reason for retention of the cluster in *A*. *kawachii* is not evident other than the species is the white koji fungus used for brewing the traditional Japanese distilled spirit shochu [43, 44]. Several different substrates are used for shochu fermentation, such as barley, cassava, buckwheat, rice, chestnut, and other ingredients that perhaps contain Bx-compounds or other similar metabolites. The lack of a *FDB2* cluster in *A*. *flavus* is interesting given the common association of this aflatoxin-producing fungus with maize. Although *A*. *flavus* is not a common endophyte or pathogen of maize vegetative tissues, the fungus is associated with plant debris in the soil [45]. While BOA-tolerance may not be essential for colonization of such debris, having the capacity to detoxify this maize phytochemical could provide fungi like *F*. *verticillioides* and *F*. *subglutinans* the opportunity to be primary colonizers that then facilitate secondary colonization by less tolerant species like *A*. *flavus* [6, 15, 36]. Further, *A*. *flavus* is a common colonizer of maize kernels, which do not produce or contain Bx-compounds [4]. Therefore, *A*. *flavus* may have little direct exposure to Bx-compounds and therefore has lacked the selective pressure to retain the *FDB2* cluster.

*Fusarium verticillioides*, *F*. *subglutinans*, and *C*. *graminicola* are all pathogens of maize, thus the host and associated phytochemicals may have exerted significant evolutionary pressure on these fungal genomes, including facilitating stable fungal-to-fungal horizontal transfer of genes (i.e., the *FDB1* cluster) that may enhance the fitness or virulence of fungi such as *C*. *graminicola*. The possession of both *FDB* clusters by *F*. *verticillioides* and *F*. *subglutinans* is not unexpected since these two species are noted for their high tolerance to BOA [5, 11]. Mbl1 encoded within the *FDB1* cluster has evolved to be the primary lactamase responsible for hydrolysis of BOA, and species lacking Mbl1, such as *F*. *pseudograminearum*, appear to utilize the Mbl2 ortholog encoded within the *FDB2* cluster [10, 23].

The presence of the *FDB1* cluster in *F*. *camptoceras* is an interesting observation that cannot be as easily rationalized as with *F*. *verticillioides* and *F*. *subglutinans*. Little is known about *F*. *camptoceras*, and it is noted primarily from decayed bananas and cacao in tropical regions [46]. Perhaps this species has a broader host range than currently recognized and includes one of the Bx-producing grass species [4], or it may encounter other compounds that are similar to Bx-compounds. We found *F*. *camptoceras* to be tolerant to BOA, but it was not as proficient at metabolizing the γ-lactam to HPMA compared to *F*. *verticillioides* and *F*. *subglutinans* (S8 Fig).

Together with the recent characterization of a γ-lactamase from *F*. *pseudograminearum* [23], these are the first detailed studies of hydrolytic lactamases in fungi. While β-lactamases are well studied in bacteria for their impact on antibiotic resistance, we have only just begun to functionally evaluate members of the metallo-β-lactamase superfamily in *Fusarium* species. We have created gene deletion mutants for many putative lactamases in *F*. *verticillioides* and are now assessing potential biological functions. In addition to identifying substrates for the encoded lactamases, future studies will address competitive interactions to evaluate the contribution of lactamases to the fitness of *F*. *verticillioides* in soils and when challenged by various naturally occurring lactam compounds. The ability to neutralize the effects of various phytochemical, microbial, and environmental xenobiotics may have contributed to the adaptive fitness and abundance of *F*. *verticillioides* in maize fields worldwide.

## Supporting Information

S1 Fig Protein sequence alignment of Mbl1 (FVEG_08291) and Mbl2 (FVEG_12637). Mbl1 has an additional 91 amino acids on its amino terminus and 62 amino acids on its carboxy terminus compared to Mbl2. Both encode the conserved protein domain of the class B metallo-β-lactamase superfamily, with the typical HxHxDHxG amino acid sequence motif (residues 147–154 in the protein sequence of Mbl1).
(TIF)

S2 Fig Deletion analysis of *AMD1*. (A) FVEG_08289 (*AMD1*) restriction map of the native and deletion alleles and Southern hybridization analysis of transformants showing the banding patterns of wild-type strain FRC M-3125 and the transformants having either homologous integration and deletion of the ORF or ectopic integration. Genomic DNA from all strains was digested with SalI (S). Wild type had a ~4.5 kb fragment whereas the deletion allele was 2.6 kb. The flank of the gene was used as probe as shown. (B) Growth of transformants on PDA amended with BOA (0.9 mg ml^-1^). The transformants are the same as assessed in (A). For example, ORF3-2 is Δ08289–2. Each transformant was evaluated in duplicate wells of the 24-well plate. Plates were incubated at 27C for 7 days.
(TIF)

S3 Fig Deletion analysis of *DLH1*. (A) FVEG_08290 (*DLH1*) restriction map of the native and deletion alleles and Southern hybridization analysis of transformants showing the banding patterns of wild-type strain FRC M-3125 and the transformants having either homologous integration and deletion of the ORF or ectopic integration. Genomic DNA from all strains was digested with HindIII (H). Wild type had a ~5.2 kb fragment whereas the deletion allele was ~3.0 kb. The 5’ flank of the gene was used as probe. (B) Growth of transformants on PDA amended with BOA (0.9 mg ml^-1^). The transformants are the same as assessed in (A). For example, ORF4-11 is Δ08290–11. Each transformant was evaluated in duplicate wells of the 24-well plate. Plates were incubated at 27C for 7 days.
(TIF)

S4 Fig HPLC assessment of 2-oxindole degradation. Uninoculated PDB containing 2-oxindole served as control. Note the difference in y-axis scale for the PDB control compared to the fungal treatments, which were wild-type FRC M-3125, the deletion mutants for *Δamd1* (Δ08289), *Δdlh1* (Δ08290), and *Δmbl1* (Δ08291), and the *Δmbl1*::*MBL1* complemented strain (Δ08291–56::C2). The smaller scale was shown for the fungal treatments to allow the peaks in those samples to be more clearly seen.
(TIF)

S5 Fig Protein based phylogeny of FVEG_08289 (*AMD1*) and FVEG_17313 (*AMD2*). The PhyML cladogram is shown with bootstrap values (200 replications) indicated for branches having ≥80% support. Bayesian posterior probabilities are also indicated for those branches. *Zymoseptoria tritici* XP_003850457 was the designated outgroup.
(TIF)

S6 Fig Protein based phylogeny of FVEG_12634. FVEG_12634 encodes a putative carboxylesterase that is not encoded within the *F*. *verticillioides FDB1* cluster, only within the *FDB2* cluster. In contrast, *F*. *camptoceras* encodes paralogs of this gene within both of its *FDB1* and *FDB2* clusters. Orthologs are present in both *C*. *graminicola* and *A*. *kawachii*, with the former grouping with the *F*. *camptoceras FDB1* cluster protein, and the latter grouping with the general *FDB2* cluster proteins. The PhyML cladogram is shown with bootstrap values (200 replications) indicated for branches having ≥80% support. Bayesian posterior probabilities are also indicated for those branches. *Zymoseptoria tritici* XP_003856069 was the designated outgroup.
(TIF)

S7 Fig Mauve analysis of the *F*. *verticillioides FDB1* cluster with the *FDB1* clusters from *F*. *subglutinans* and *F*. *camptoceras*, along with the homologous cluster from *C*. *graminicola*. Mauve identifies locally collinear blocks (LCBs), which are conserved genomic segments that appear to be internally free of rearrangements. Each LCB is a different color. Genes from each cluster are depicted. Conserved intron-exon structure can be observed among the orthologs. The *MBL1* orthologs are noted with the cursor. (A) Highly conserved synteny and LCB of *FDB1* gene clusters from *F*. *verticillioides*, *F*. *subglutinans*, and *C*. *graminicola*. (B) Synteny comparison of *F*. *verticillioides FDB1* gene cluster with that of *F*. *camptoceras*. (C) Synteny comparison of the *C*. *graminicola* gene cluster with the *FDB1* gene cluster of *F*. *camptoceras*. (D) Comparison of all four fungi. Note that the *F*. *camptoceras MBL1* is inverted in relation to the *MBL1* from the other fungi.
(TIF)

S8 Fig TLC assessment of BOA metabolism to HPMA. The metabolism of BOA was assessed by thin-layer chromatography (TLC) after 7-days incubation of the fungi on BOA medium (1.0 mg ml^-1^) in standard 100 x 15 mm plates. Agar plugs were taken from just beyond the colony margin of each fungus and spotted onto TLC sheets to apply extracellular metabolites. The ability of strains to metabolize BOA to HPMA was compared to *F*. *verticillioides* wild-type strain FRC M-3125. Two strains of *F*. *subglutinans*, NRRL 22002 and NRRL 22034, were able to convert BOA to HPMA as effectively as *F*. *verticillioides*. *Fusarium camptoceras* NRRL 13381 along with two other isolates tentatively identified as *F*. *camptoceras*, NRRL 20697 and NRRL 20716, did not produce HPMA to the same degree as *F*. *verticillioides* and *F*. *subglutinans*. Similar results were evident for *F*. *oxysporum* NRRL 34936 and *F*. *graminearum* NRRL 31084. BOA standard (10 and 100 μg) was applied in the first and last positions on the TLC sheet, and HPMA standard (5 μg) was applied in the second position.
(TIF)

S9 Fig Map indicating the relative location of primers used in the creation and validation of FVEG_08289 (*AMD1*), FVEG_08290 (*DLH1*), and FVEG_08291 (*MBL1*) deletions. Refer to S2 Table for the primer sequences.
(TIF)

S1 Table Microarray analysis of *F*. *verticillioides* increased gene expression in response to BOA exposure identified the *FDB1* and *FDB2* gene clusters. (PDF)

S2 Table Primers used in this study. (DOCX)
