# Supplementary figures and images for: Two Horizontally Transferred Xenobiotic Resistance Gene Clusters Associated with Detoxification of Benzoxazolinones by Fusarium Species
Source: PLoS One. 2016 Jan 25;11(1):e0147486. doi: 10.1371/journal.pone.0147486 (PMC4726666; doi:10.1371/journal.pone.0147486)

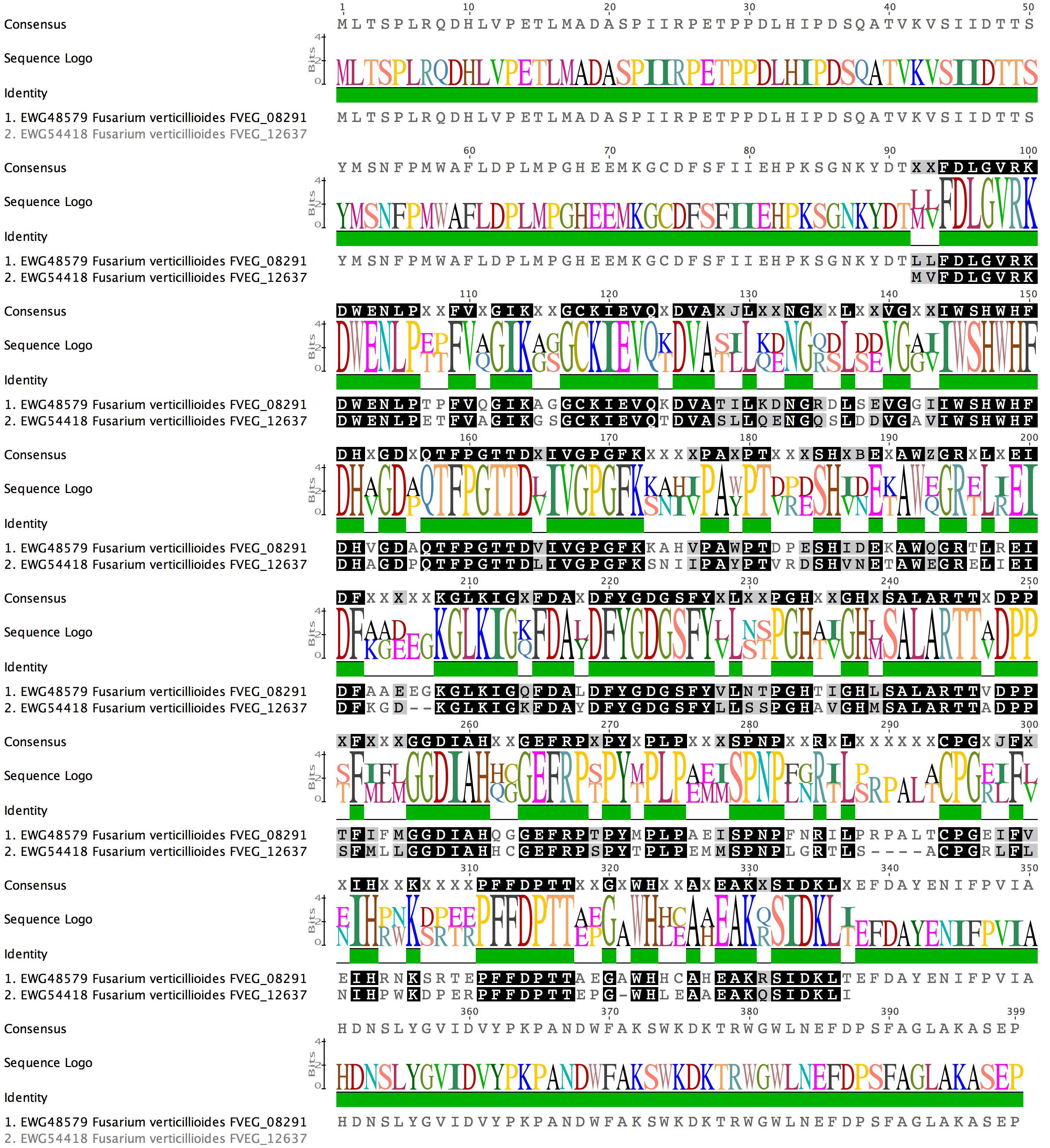

Supplement: S1 Fig — Mbl1 has an additional 91 amino acids on its amino terminus and 62 amino acids on its carboxy terminus compared to Mbl2. Both encode the conserved protein domain of the class B metallo-β-lactamase superfamily, with the typical HxHxDHxG amino acid sequence motif (residues 147–154 in the protein sequence of Mbl1). (TIF) [file pone.0147486.s001.tif]

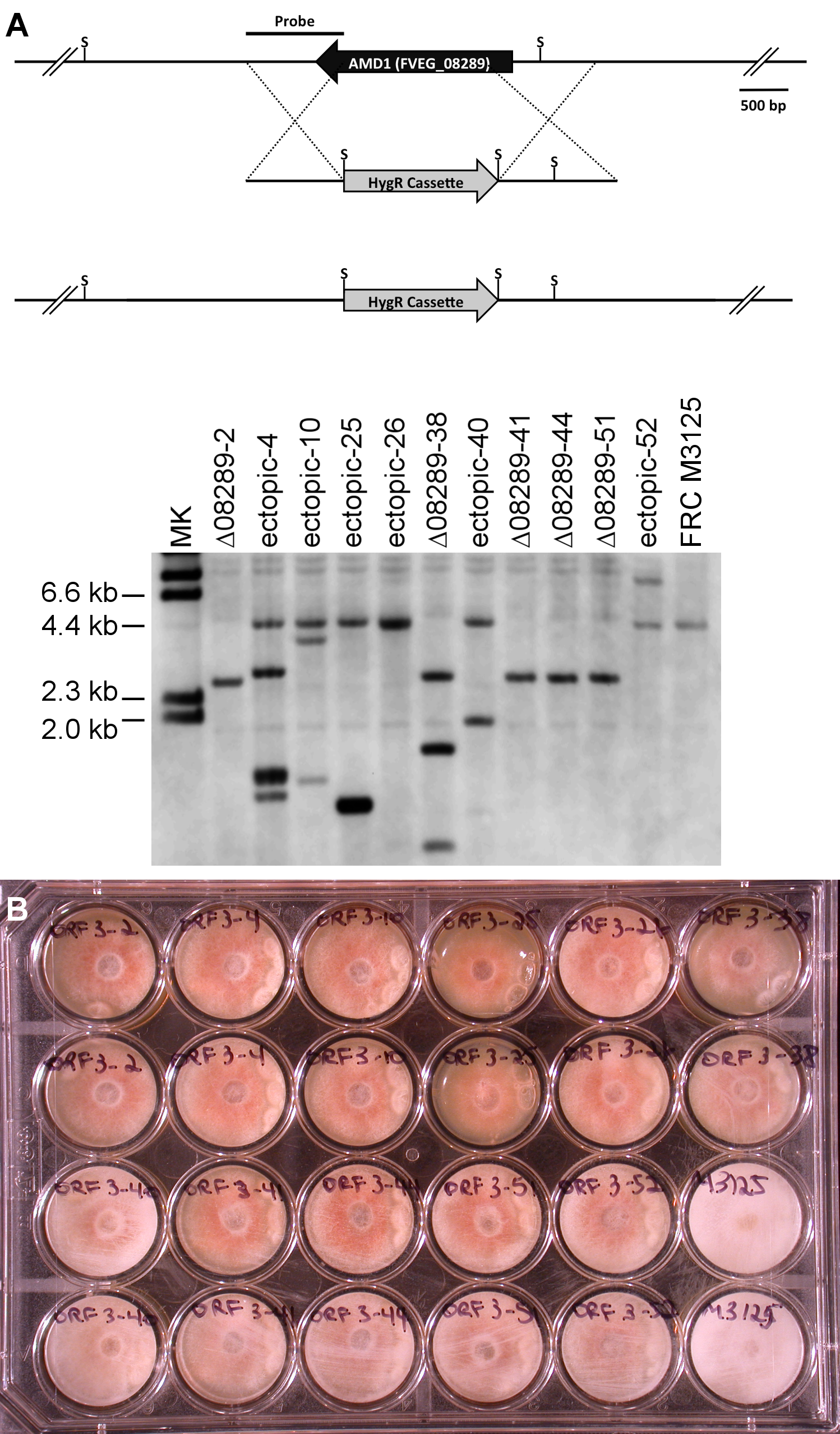

Supplement: S2 Fig — (A) FVEG_08289 (AMD1) restriction map of the native and deletion alleles and Southern hybridization analysis of transformants showing the banding patterns of wild-type strain FRC M-3125 and the transformants having either homologous integration and deletion of the ORF or ectopic integration. Genomic DNA from all strains was digested with SalI (S). Wild type had a ~4.5 kb fragment whereas the deletion allele was 2.6 kb. The flank of the gene was used as probe as shown. (B) Growth of transformants on PDA amended with BOA (0.9 mg ml-1). The transformants are the same as assessed in (A). For example, ORF3-2 is Δ08289–2. Each transformant was evaluated in duplicate wells of the 24-well plate. Plates were incubated at 27C for 7 days. (TIF) [file pone.0147486.s002.tif]

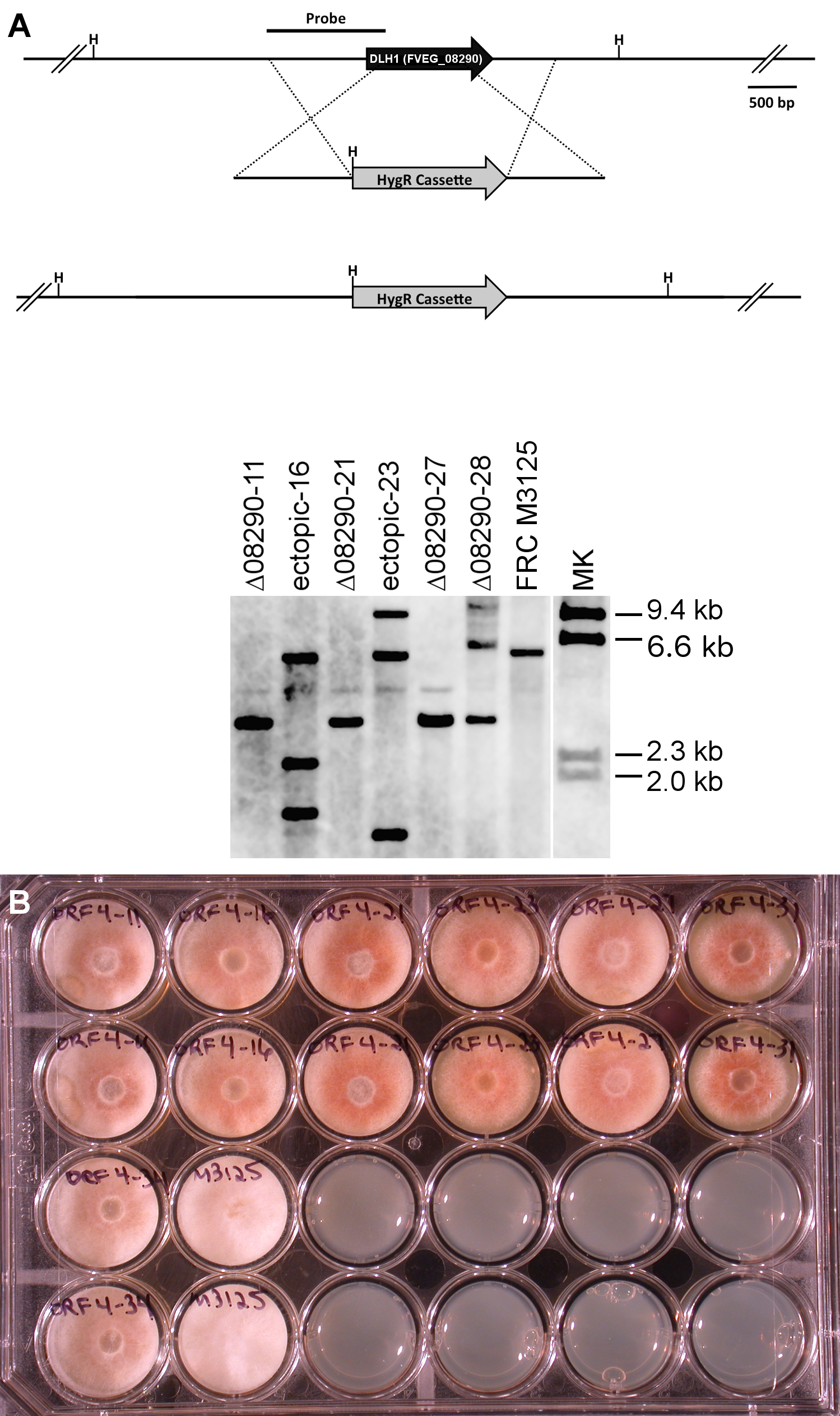

Supplement: S3 Fig — (A) FVEG_08290 (DLH1) restriction map of the native and deletion alleles and Southern hybridization analysis of transformants showing the banding patterns of wild-type strain FRC M-3125 and the transformants having either homologous integration and deletion of the ORF or ectopic integration. Genomic DNA from all strains was digested with HindIII (H). Wild type had a ~5.2 kb fragment whereas the deletion allele was ~3.0 kb. The 5’ flank of the gene was used as probe. (B) Growth of transformants on PDA amended with BOA (0.9 mg ml-1). The transformants are the same as assessed in (A). For example, ORF4-11 is Δ08290–11. Each transformant was evaluated in duplicate wells of the 24-well plate. Plates were incubated at 27C for 7 days. (TIF) [file pone.0147486.s003.tif]

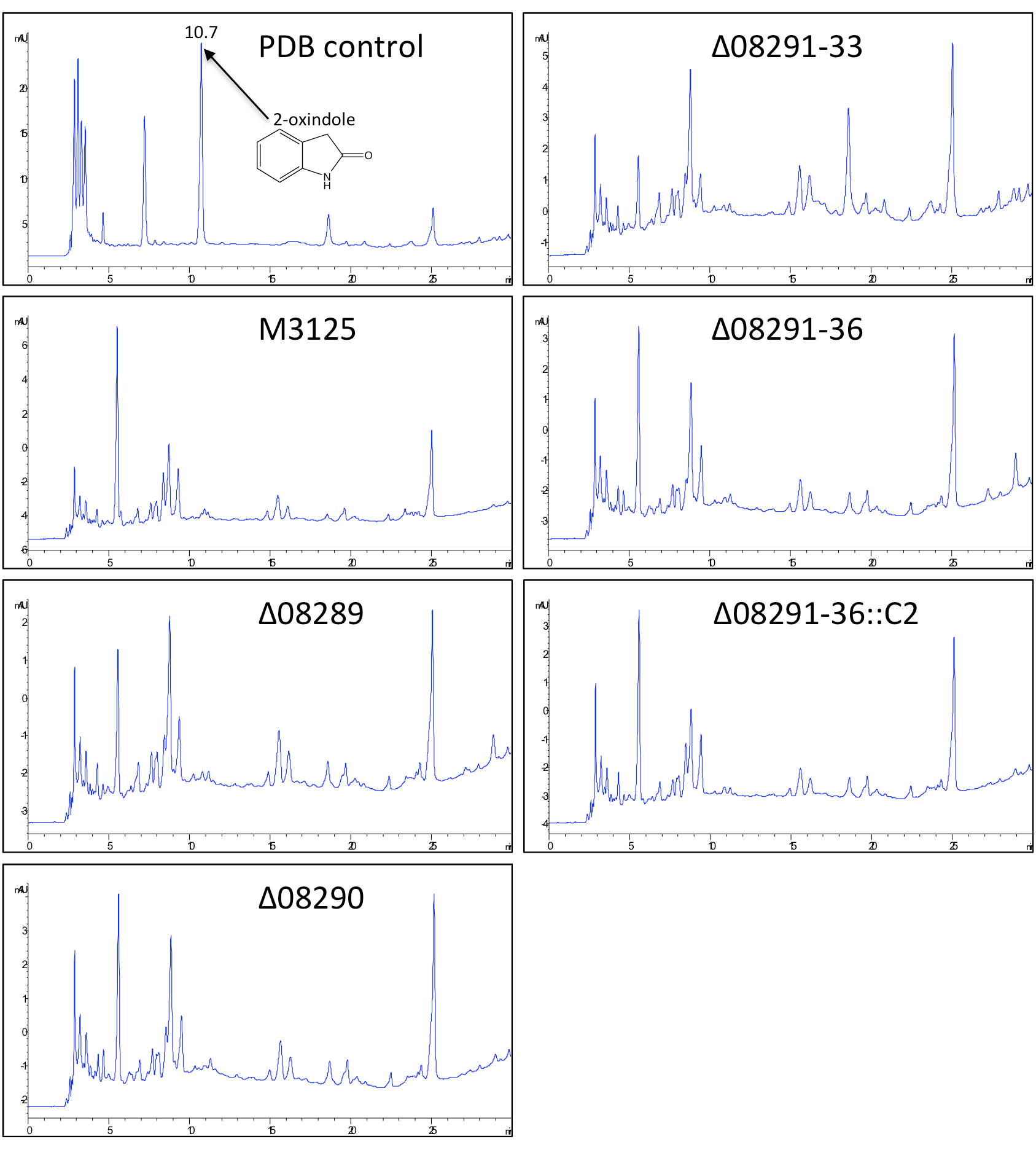

Supplement: S4 Fig — Uninoculated PDB containing 2-oxindole served as control. Note the difference in y-axis scale for the PDB control compared to the fungal treatments, which were wild-type FRC M-3125, the deletion mutants for Δamd1 (Δ08289), Δdlh1 (Δ08290), and Δmbl1 (Δ08291), and the Δmbl1::MBL1 complemented strain (Δ08291–56::C2). The smaller scale was shown for the fungal treatments to allow the peaks in those samples to be more clearly seen. (TIF) [file pone.0147486.s004.tif]

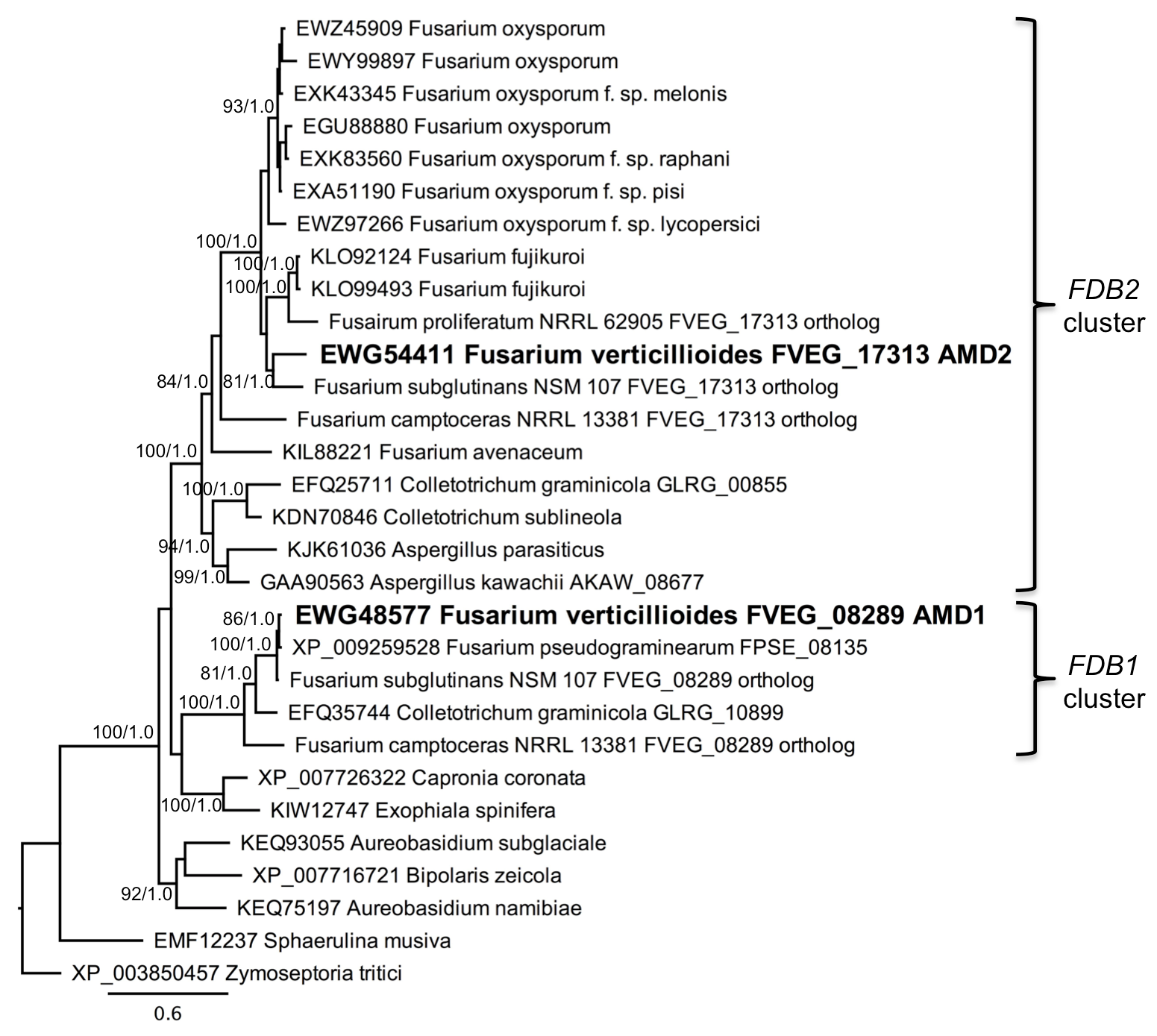

Supplement: S5 Fig — The PhyML cladogram is shown with bootstrap values (200 replications) indicated for branches having ≥80% support. Bayesian posterior probabilities are also indicated for those branches. Zymoseptoria tritici XP_003850457 was the designated outgroup. (TIF) [file pone.0147486.s005.tif]

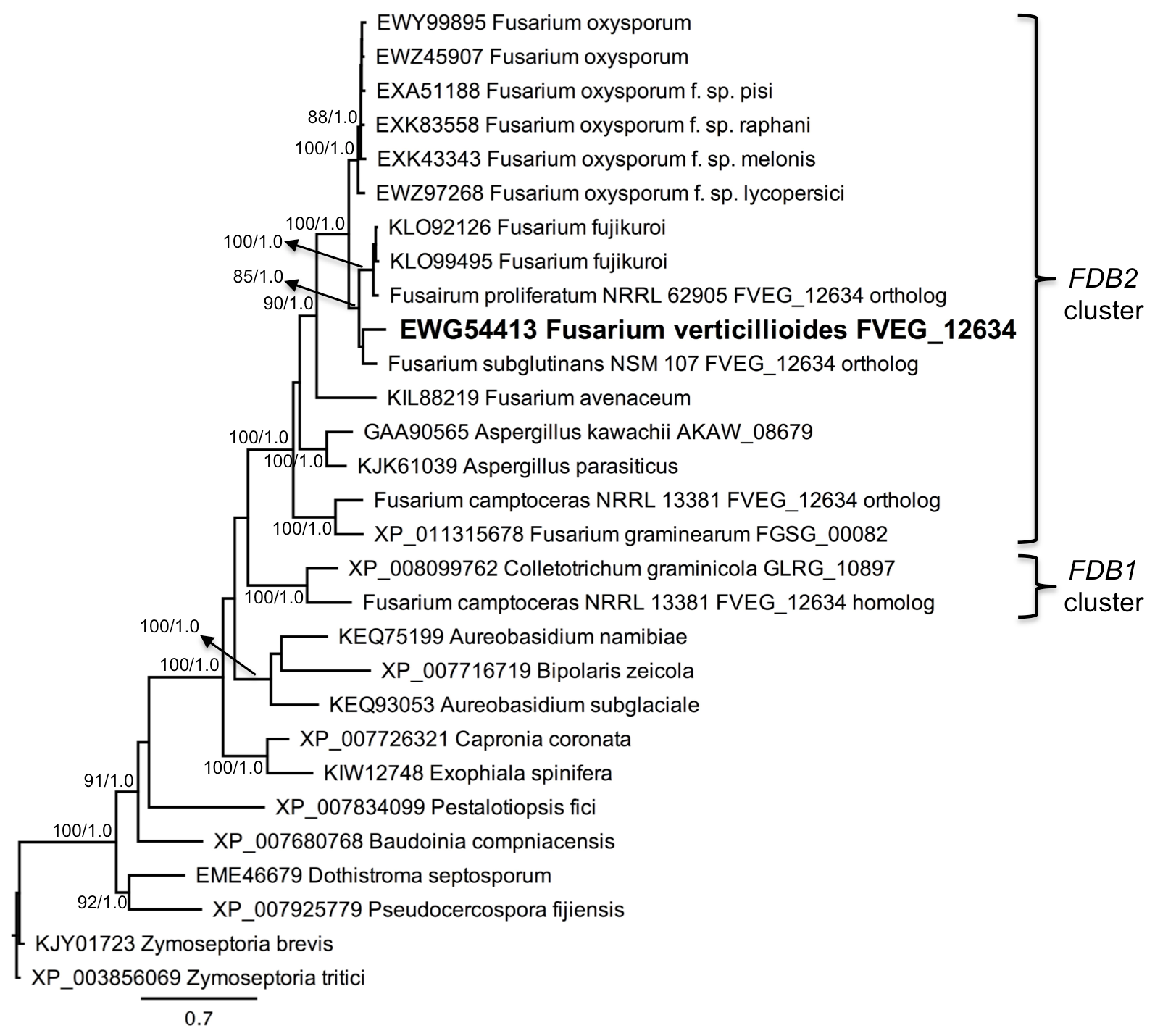

Supplement: S6 Fig — FVEG_12634 encodes a putative carboxylesterase that is not encoded within the F. verticillioides FDB1 cluster, only within the FDB2 cluster. In contrast, F. camptoceras encodes paralogs of this gene within both of its FDB1 and FDB2 clusters. Orthologs are present in both C. graminicola and A. kawachii, with the former grouping with the F. camptoceras FDB1 cluster protein, and the latter grouping with the general FDB2 cluster proteins. The PhyML cladogram is shown with bootstrap values (200 replications) indicated for branches having ≥80% support. Bayesian posterior probabilities are also indicated for those branches. Zymoseptoria tritici XP_003856069 was the designated outgroup. (TIF) [file pone.0147486.s006.tif]

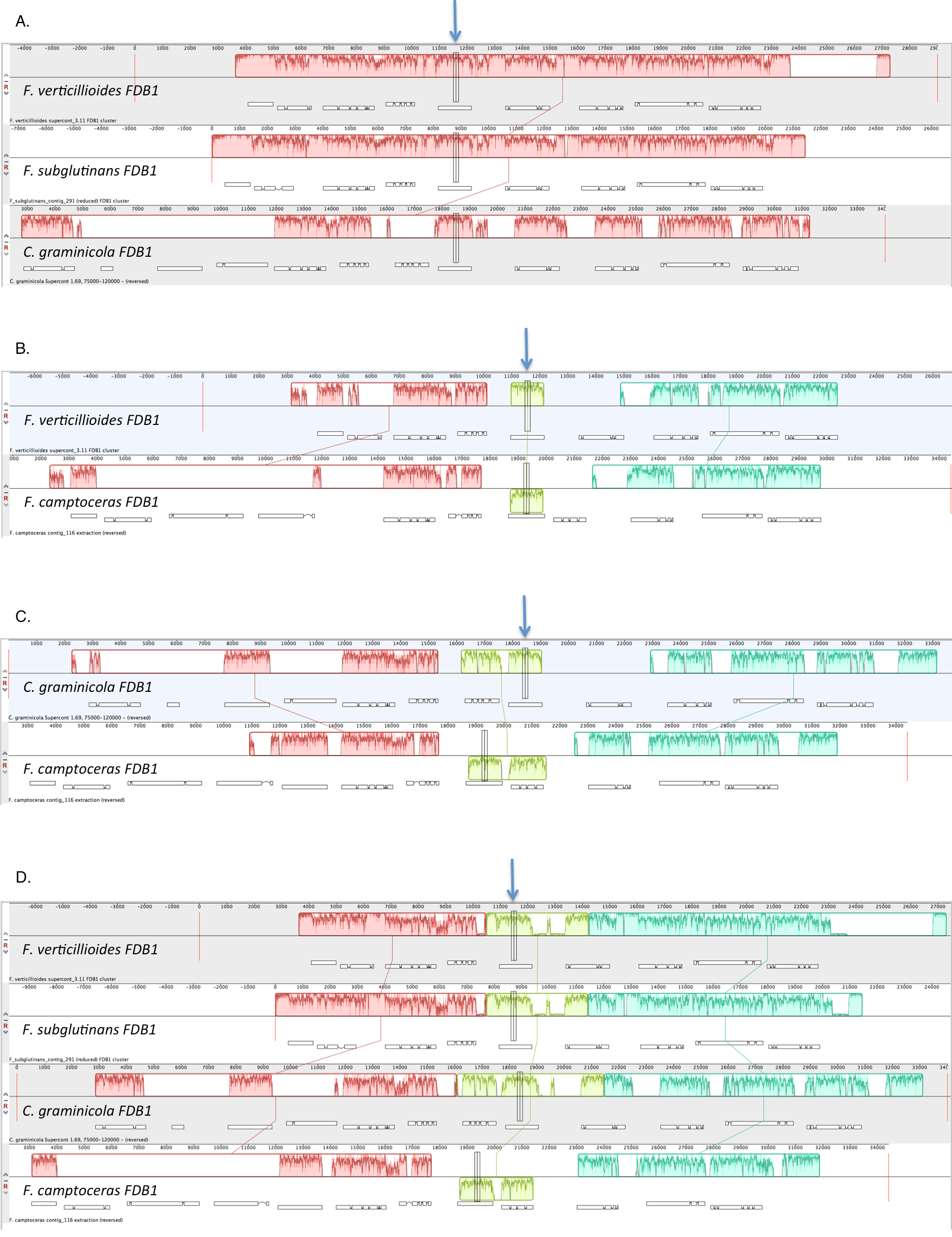

Supplement: S7 Fig — Mauve identifies locally collinear blocks (LCBs), which are conserved genomic segments that appear to be internally free of rearrangements. Each LCB is a different color. Genes from each cluster are depicted. Conserved intron-exon structure can be observed among the orthologs. The MBL1 orthologs are noted with the cursor. (A) Highly conserved synteny and LCB of FDB1 gene clusters from F. verticillioides, F. subglutinans, and C. graminicola. (B) Synteny comparison of F. verticillioides FDB1 gene cluster with that of F. camptoceras. (C) Synteny comparison of the C. graminicola gene cluster with the FDB1 gene cluster of F. camptoceras. (D) Comparison of all four fungi. Note that the F. camptoceras MBL1 is inverted in relation to the MBL1 from the other fungi. (TIF) [file pone.0147486.s007.tif]

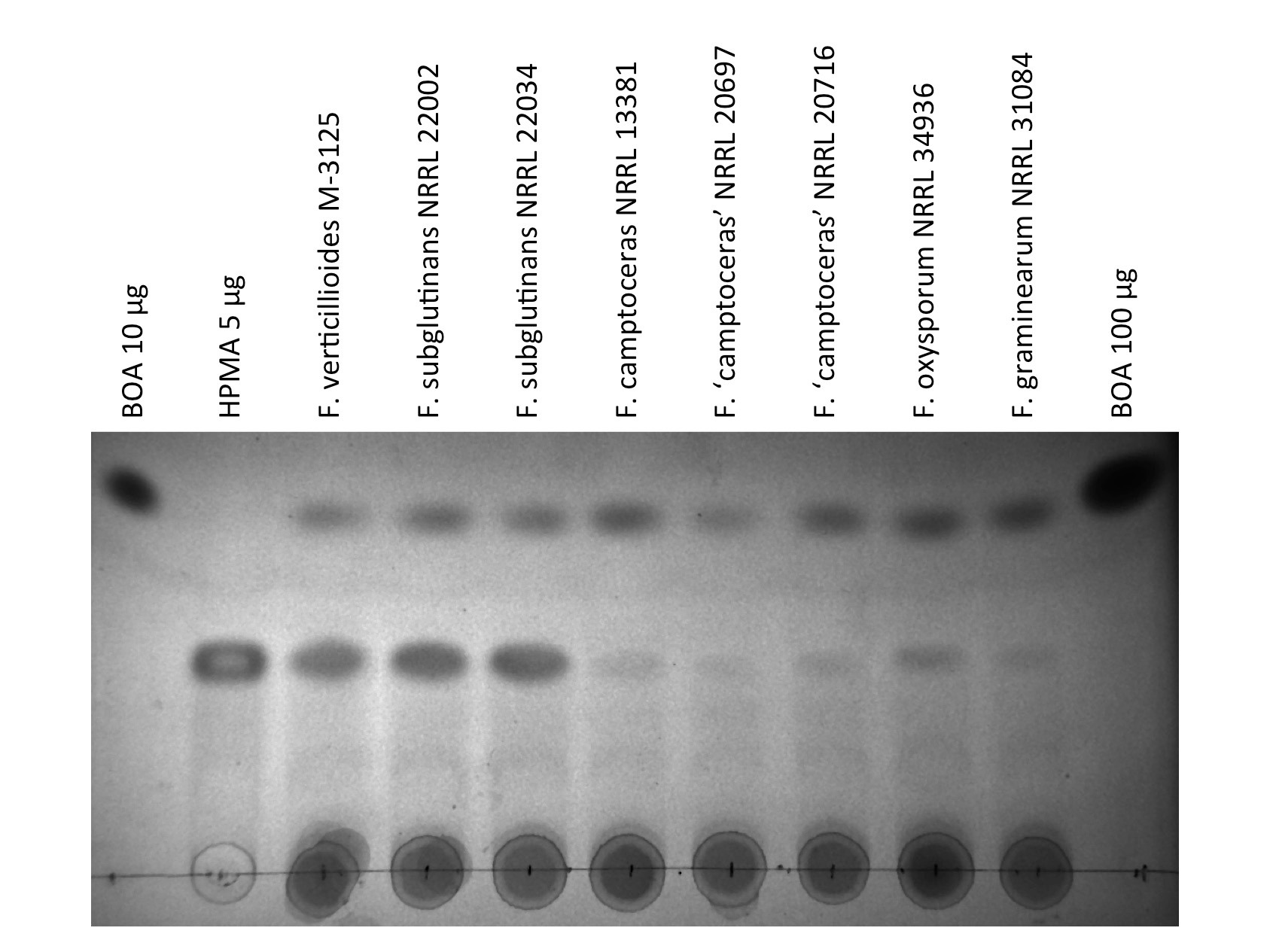

Supplement: S8 Fig — The metabolism of BOA was assessed by thin-layer chromatography (TLC) after 7-days incubation of the fungi on BOA medium (1.0 mg ml-1) in standard 100 x 15 mm plates. Agar plugs were taken from just beyond the colony margin of each fungus and spotted onto TLC sheets to apply extracellular metabolites. The ability of strains to metabolize BOA to HPMA was compared to F. verticillioides wild-type strain FRC M-3125. Two strains of F. subglutinans, NRRL 22002 and NRRL 22034, were able to convert BOA to HPMA as effectively as F. verticillioides. Fusarium camptoceras NRRL 13381 along with two other isolates tentatively identified as F. camptoceras, NRRL 20697 and NRRL 20716, did not produce HPMA to the same degree as F. verticillioides and F. subglutinans. Similar results were evident for F. oxysporum NRRL 34936 and F. graminearum NRRL 31084. BOA standard (10 and 100 μg) was applied in the first and last positions on the TLC sheet, and HPMA standard (5 μg) was applied in the second position. (TIF) [file pone.0147486.s008.tif]

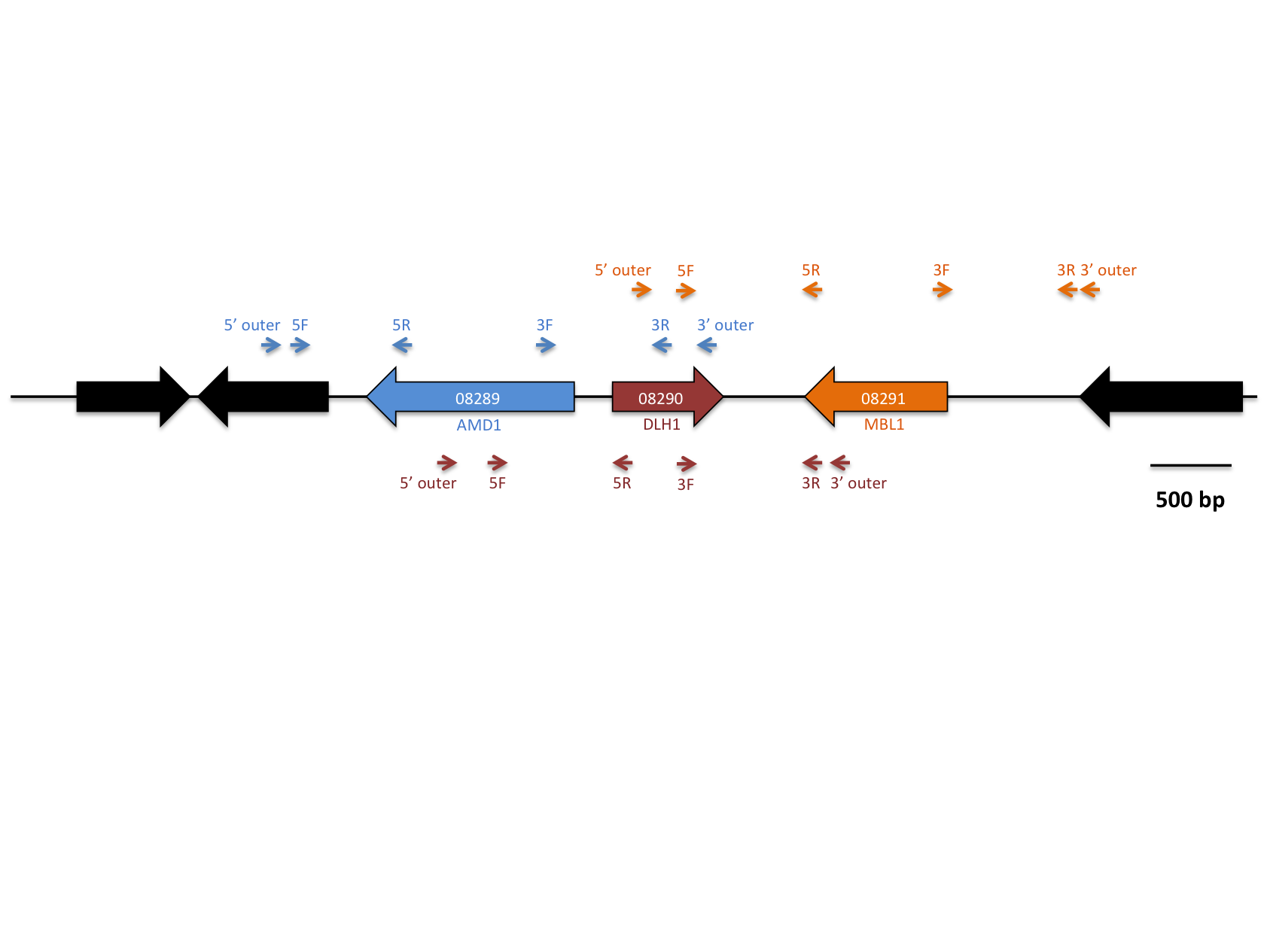

Supplement: S9 Fig — Refer to S2 Table for the primer sequences. (TIF) [file pone.0147486.s009.tif]
