# Supplementary material for: Two Horizontally Transferred Xenobiotic Resistance Gene Clusters Associated with Detoxification of Benzoxazolinones by Fusarium Species
Source: PLoS One. 2016 Jan 25;11(1):e0147486. doi: 10.1371/journal.pone.0147486 (PMC4726666; doi:10.1371/journal.pone.0147486)
Supplement: S1 Table — (PDF) [file pone.0147486.s010.pdf]

S1 Table. Microarray analysis of *F. verticillioides* increased gene expression in response to BOA exposure identified the *FDB1* and *FDB2* gene clusters.[a]

FVEG Locus	Putative function based on BLASTP and conserved domain searches	Avg Fold Change
FVEG_05718	Major facilitator superfamily transporter	7.9
FVEG_05719	M20 Peptidase beta-alanine synthase, an amidohydrolase	5.5
FVEG_07606	Acyl coenzyme A transferase	3.1
FVEG_07761	Sugar porter subfamily of the major facilitator superfamily	3.8
FVEG_07762	Beta-glucosidase-related glycosidase	3.2
FVEG_13837	Benzyl alcohol dehydrogenase	0.9
FVEG_13838	Zn(II)2Cys6 transcription factor	1.1
FVEG_08287	NmrA-like family	3.0
FVEG_08288	Major facilitator superfamily transporter	5.2
FVEG_08289	Amidase	23.3
FVEG_08290	Dienelactone hydrolase	37.9
FVEG_08291	Metallo-β-lactamase superfamily	12.9
FVEG_08292	Amino acid transporter	42.6
FVEG_08293	Salicylate hydroxylase	9.8
FVEG_08294	Zn(II)2Cys6 transcription factor	4.4
FVEG_08295	Amidase	21.5
FVEG_08296	Major facilitator superfamily transporter	1.2
FVEG_08297	Cation efflux family	1.0
FVEG_08792	SRR1-like protein possibly involved in circadian regulation	3.3
FVEG_09100	Extradiol ring-cleavage dioxygenase class III enzyme subunit B	3.5
FVEG_12624	Hypothetical protein	1.0
FVEG_12625	Dienelactone hydrolase	4.9
FVEG_12626	Major facilitator superfamily transporter	1.0
FVEG_12627	Hypothetical protein	0.9
FVEG_12628	3-Hydroxylacyl-CoA dehydrogenase	2.2
FVEG_12629	CoA-transferase family III (CaiB/BaiF)	8.1
FVEG_12630	Salicylate hydroxylase	1.6
FVEG_12631	Amidase	1.0
FVEG_12632		1.0
FVEG_12633	Malonyl-CoA synthetase	14.0
FVEG_12634	Carboxylesterase	10.0
FVEG_12635	Zn(II)2Cys6 transcription factor	5.5
FVEG_12636	Arylamine N-acetyltransferase (NAT1)	20.3
FVEG_12637	Metallo-β-lactamase superfamily	1.9
FVEG_12638	Aldo/keto reductase	20.4
FVEG_12639	Alpha/beta hydrolase; lipase	9.5
FVEG_12640	Amino acid transporter	8.9
FVEG_12641	Phthalate dioxygenase reductase	26.5

FVEG_12642	NmrA-like family	1.0
FVEG_13748	Amino acid permease	2.7
FVEG_13749	Copper amine oxidase	4.5
FVEG_14165	CoA-transferase family III (CaiB/BaiF)	11.9

[a] Of the 28 genes having ≥3-fold increase in expression, 9 were in *FDB1* (FVEG_08287 to 08295) and 10 were in *FDB2* (FVEG_12625 to 12641). Both clusters are shaded grey, with uninduced genes flanking the clusters included for comparative purposes. Note that FVEG_12631 and 12632 were reannotated and designated FVEG_17313, as indicated in Figure 2. Also, a gap exists in the available sequence of chromosome 10 between FVEG_13838 and FVEG_08287, which are on different contigs, thus we assume these two genes are directly adjacent to each other.
