# Supplementary material for: Two Horizontally Transferred Xenobiotic Resistance Gene Clusters Associated with Detoxification of Benzoxazolinones by Fusarium Species
Source: PLoS One. 2016 Jan 25;11(1):e0147486. doi: 10.1371/journal.pone.0147486 (PMC4726666; doi:10.1371/journal.pone.0147486)
Supplement: S2 Table — (DOCX) [file pone.0147486.s011.docx]

**S2 Table. Primers used in this study.**

| **Primer^a^** | **Sequence (5’—3’)^b^** |
| --- | --- |
| 1B8For | CCCATCAGAGAACAAACTGTCTAA |
| 4B3For | ATCAAAATCATGGATGAGCATTAC |
| 4B3Rev | AAAGATAGCGCACTGTAGTCGAG |
| FvAm4^c^ | TCCTTGTGCCTACTATGCCG |
| FvAm18^d^ | CAATCTGGATGATGCTTCC |
| HygFor | TCGTGCTTTCAGCTTCGATGTAGG |
| HygRev | CATTGTCCGTCAGGACATTGTTGG |
| M13Fv2 | CGCCAGGGTTTTCCCAGTCACGAC |
| M13Rv2 | AGCGGATAACAATTTCACACAGGA |
| AMD1-5’outer | CAATGTTGAGGATGTAGACG |
| AMD1-5F | GTTGTAACTGAGTGGACAGATG |
| AMD1-5R | TCCTGTGTGAAATTGTTATCCGCTTCCTTGTGCCTACTATGCCG |
| AMD1-3F | GTCGTGACTGGGAAAACCCTGGCGATGTGAGCAAGGACAACGG |
| AMD1-3R | GTTCACAAAAGATGGGTGG |
| AMD1-3’outer | AGCCACTCAATGAATCCC |
| AMD1-qPCRfor | AGTGCGCTATCTTTCCCAGCA |
| AMD1-qPCRrev | ATCCGCCGACCCACAATCTGTA |
| DLH1-5’outer | TGGCGGATGAACCTTTAC |
| DLH1-5F | CCCAAAGTGGATTATCTGTTTCTC |
| DLH1-5R | TCCTGTGTGAAATTGTTATCCGCTCAATCTGGATGATGCTTCC |
| DLH1-3F | GTCGTGACTGGGAAAACCCTGGCGGATATGCACAGAGAAGCAGC |
| DLH1-3R^e^ | AAGGTCGCTGATTTCGATGG |
| DLH1-3’outer | AGAGTTGGAAGGACAAGACCAG |
| DLH1-qPCRfor | TCTCAAATGAGGCTCGCACA |
| DLH1-qPCRrev | TCCCTTGAAATGCTGCTCCT |
| MBL1-5’outer | CAAACACCTTCTTCCCTC |
| MBL1-5F | GATATGCACAGAGAAGCAGC |
| MBL1-5R | GTCGTGACTGGGAAAACCCTGGCGAAGGTCGCTGATTTCGATGG |
| MBL1-3F | TCCTGTGTGAAATTGTTATCCGCTTGTGTCTTGGACGAATCGGA |
| MBL1-3R | AAGGAGCCGTTTGGACATCA |
| MBL1-3’outer | CGAATCTATCTTTACGCGGCGA |
| MBL1for | TGGGCATCACCAACATGGTCAA |
| MBL1rev | AAGGCCTTTGGTGCTTGCGTTT |
| MBL1-qPCRfor | AAACGCAGCATCGACAAGCTGA |
| MBL1-qPCRrev | TAGGGTCGAACTCATTGAGCCA |

^a^ Refer to S9 Fig for location of some of the primers.

^b^ The underlined sequence in AMD1-5R, DLH1-5R, and MBL1-3F is the reverse complement of primer M13Rv2, and the underlined sequence in AMD1-3F, DLH1-3F, and MBL1-5R is the reverse complement of primer M13Fv2.

^c^ FvAm4 is the same sequence as the gene-specific region of primer AMD1-5R.

^d^ FvAm18 is the same sequence as the gene-specific region of primer DLH1-5R.

^e^ DLH1-3R is the same sequence as the gene-specific region of primer MBL1-5R.
